# Equations of Disturbed Motion of the Moving Part of the Gyroscope Suspension

**DOI:** 10.3390/s22197442

**Published:** 2022-09-30

**Authors:** Igor Korobiichuk, Viktorij Mel’nick, Vera Kosova, Kateryna Maksymenko

**Affiliations:** 1 Łukasiewicz Research Network—Industrial Research Institute for Automation and Measurements PIAP, 02-486 Warsaw, Poland; 2 Faculty of Biotechnology and Biotechnics, National Technical University of Ukraine “Igor Sikorsky Kyiv Polytechnic Institute”, 03056 Kyiv, Ukraine

**Keywords:** inertial sensors, gyroscope, mathematical model, launch vehicle, suspension, acoustic radiation

## Abstract

The response of the float two-stage angular velocity sensor to the simultaneous perturbation from the rocket body—kinematic perturbation—and the penetrating acoustic radiation from the propulsion engines of the launch vehicle were determined. The solution of two equations was successively analyzed: the first and second approximations, and the synchronous and asynchronous fuselage pitch. The reaction of the float gyroscope to harmonic oscillations of the base was analyzed. The effect of the zero shift of the device due only to the angular oscillations of the launch vehicle body and the penetrating acoustic radiation was considered. The presented results reveal the nature of the appearance of inertia forces acting on the impedance surface of the gyroscope float suspension. Acoustic radiation that passes into a device generates many vibration modes on the surface and can have a considerable effect on the precision of float two-stage angular velocity sensor and gyro-stabilized platforms.

## 1. Introduction

The development of hypersonic technology creates a new challenge for inertial navigation sensors, which are widely used in aviation navigation systems [1]. The operation of sensors that are part of navigation systems, such as gyroscopes, work in difficult conditions, which affects their accuracy [2,3].

The differentiating gyroscopes are arranged in one unit—a rate-gyro unit—and oriented with their four sensitivity axes by the three axes of fuselage. The integrating gyroscope’s float gimbal is often used as a sensitive element in the three-axial gyro-stabilized platform [4,5].

The papers [6] analyzed the possibility of occurrence of the constant constituents in the analytic representation of the moments-interferences caused by the diffractional phenomena of the penetrative acoustic radiation. The availability of a constant constituent of arising moment-interference will lead to the systematic exit of the gyroscope with respect to the output axis and, consequently, to the systematic platform’s wandering with respect to the axes of stabilization [7].

**Figure 1:**
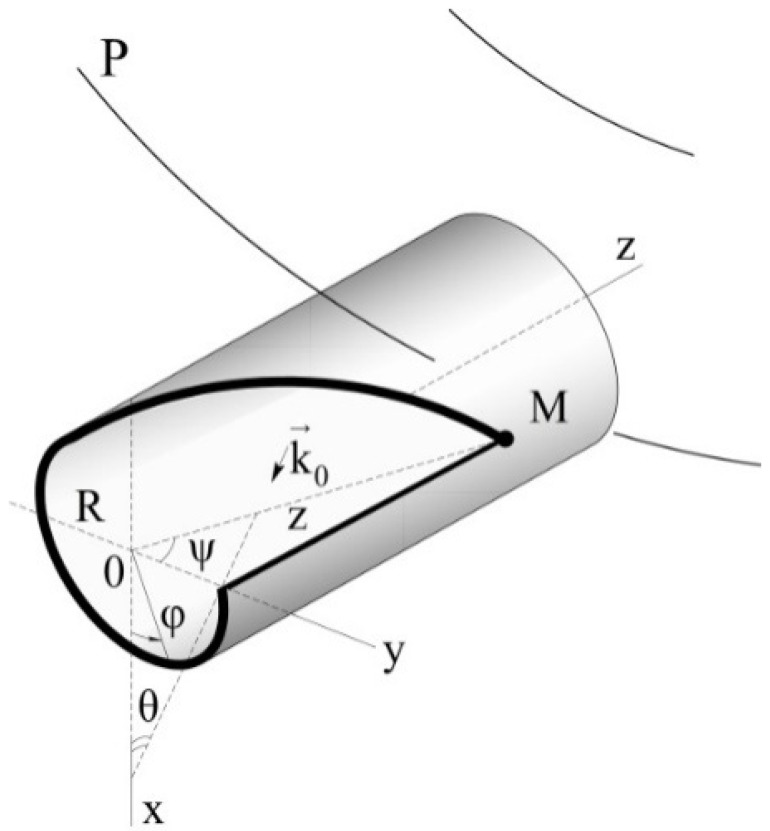
Disturbing factors as the result of the diffractional effects on the gyroscope gimbal’s impedance surface.

The most susceptible to the flying vehicle’s angular motion are the elastic displacements of the gimbal’s surface in the plane of frame [8]. The hypothesis has logic justification and explores considerable (approximately by two orders) exceedance of the amplitude of elastic displacements in this direction in comparison with two others [9]. The gimbal’s elastic stress interacts with all three constituents of the vehicle’s angular motion and serves for the occurrence of disturbing factors acting on the gimbal’s output axis (Figure 1). Thus, the angular velocity contributes to the occurrence of the angular acceleration.

The occurrence of additional inaccuracies of the float gimbal in acoustic fields and establishing the extent of influence of each of the disturbing factors was analyzed in the papers [10,11,12].

When acoustic pressures are at 150 dB and more, the gimbal’s surface changes from absolutely solid to the impedance one, i.e., on the surface of which, under the effect of force impact from the penetrative radiation, there are elastic displacements of surface that arise in three directions—along the float’s length (coordinate), circumferentially (along the parallel, coordinate), and transversally (in the plane of frame, coordinate) [13,14].

Till the gimbal’s surface can be considered as absolutely solid, all the float’s properties are concentrated in one parameter—inertia moment. The mechanism for the device’s inaccuracy is described from here on [15].

If the surface turns into the impedance one and performs induced elastic motions, in virtue of the gyroscope’s inertial properties, the gimbal’s elastic stress will be perceived by the device as an input value and generate an additional inaccuracy of measurements represented by a reaction to a “false” angular velocity of the flying vehicle’s body. Since the float gimbal’s surface is quite large, the integral inaccuracy will also reach considerable values. Herewith, its periodic components are not so much dangerous as the systematic components which take place [16,17].

Profound studying of the phenomenon [6] confirmed the hypothesis that the elastic displacements of the gimbal’s surface are not as undesirable as the aggregate simultaneous action on the device of the kinematic disturbance on the part of the flying vehicle’s body in the form of its angular motion and elastic displacements of the gimbal’s surface under the influence of transmitted acoustic radiation and diffractional effects caused by it. Moreover, it appropriately alerts the fact that a liquid and static gimbal is a perfect conductor of sound waves and in no way serves for the energy dispersion of the transmitted field. As a result, the very idea of the float gimbal under these conditions goes to the shaky ground of unreliability of the air-borne equipment under the operational use of hypersonic flying vehicles [18,19].

Another danger for the inertial navigation devices that need to be pointed out is multicycle loading; this takes place, for example, in long-action flying vehicles [20]. The acoustic inaccuracy of the float-integrating gyroscope in this case will be summing up all the time and, all in all, may lead to emergency situations [21]. This is particularly the case when these devices are sensitive elements of the three-axial gyro-stabilized platform of electro-optical equipment, infra-red homing optical heads, gyrocompasses, and gyro-theodolites [22,23,24,25].

The aforementioned suggest the idea that, for acoustic fields, the existing calculated models of gyroscopes’ inaccuracies require critical and comprehensive reinterpretation [9]. First of all, the gimbal should be considered as a system with distributed parameters or discrete-continuous parameters. In addition, one has to take into account the inalterably present fuselage’s swinging. Such an approach corresponds to the fullest extent with the available realities of the full-scale conditions.

In this fashion, the gyroscope gimbal’s length commensurability with half of the penetrative acoustic radiation’s wavelength should be considered as a starting point when calculation models’ development [6,11,15].

In this fashion, displacements of the float gimbal’s surface under the action of penetrative acoustic radiation on the swinging base form elastic stress of the gimbal, which is perceived by the gyroscope as the input value, the angular velocity with respect to the axis *z* (Figure 1). False angular velocity can contain both periodic constituents and the systematic constituent [6].

The purpose of this research is to analyze the possibility of a systematic zero shift of the gyroscope during asynchronous pitching of the fuselage in flight conditions. The analysis of measurements’ inaccuracy will be conducted from the perspective of the mutual, simultaneous impact of two perturbing factors—kinematic and penetrative acoustic radiation of high level, namely 150 dB, and more external disturbances. We proceeded from the premise that it should be not only constantly present factors but also the typical ones for the whole class of products—controllable flying vehicles, unmanned flying vehicles, remotely controllable, disc wing aircraft, tactical carrier-based aircraft, strategic bombers aircraft, cruise missiles of various modifications and basing.

## 2. Materials and Methods

Let us write down the differential equation of the perturbed motion of the device in the form [21]:

(1) $$B\ddot{\beta }+R\{[{\left(\omega_{z}+\omega_{2}^{a}\cos\beta \right)}^{2}-\omega_{x}^{2}]\sin\beta \cos\beta -\omega_{x}\left(\omega_{z}+\omega_{2}^{a}\cos\beta \right)\cos2\beta \}\;+H\left[\omega_{x}\sin\beta +\left(\omega_{z}+\omega_{2}^{a}\cos\beta \right)\cos\beta \right]+B\left({\dot{\omega }}_{y}+{\dot{\omega }}_{1}^{a}\right)+c\beta +b\dot{\beta }=0$$

where $B=I_{0}+I_{y}$; $R=I_{0}+I_{z}-I_{x}$; $I_{x},I_{y},I_{z}$—moments of float inertia; $I,I_{0}$—polar and equatorial moments of rotor inertia; c,b—the spring stiffness factor and the damping factor, respectively, and *H*—kinetic moment of the gyroscope.

**Figure 2:**
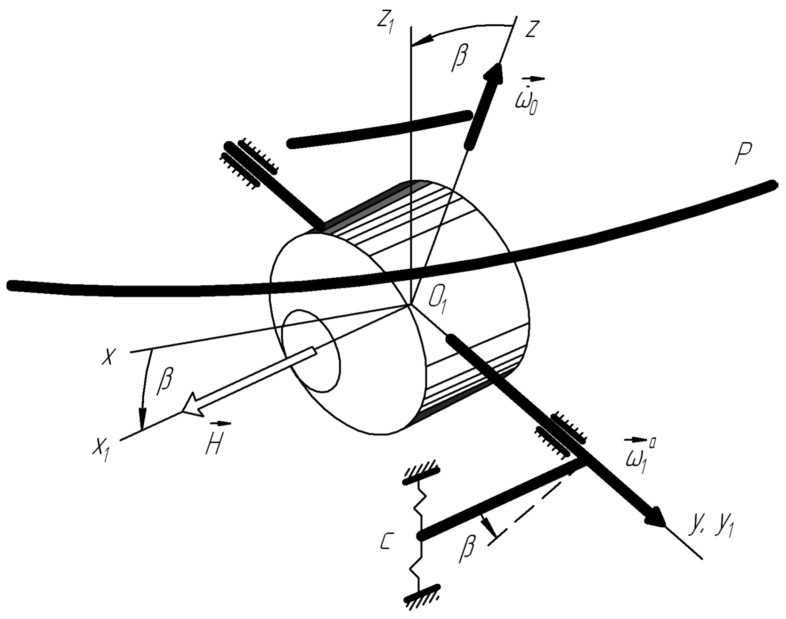
The nature of the appearance of additional acceleration ${\dot{\omega }}_{1}^{a}\left(t\right)$. due to the elastic interaction of the float surface with acoustic radiation on a pitching baseplate.

Consider a special case. Let $\omega_{x}=\omega_{y}=0$, $\omega_{z}=\omega_{0}=const$ and acoustic pressure $P_{0}=const$. It is easy to establish a relationship between the established value of the float rotation angle $\beta_{0}$, the angular velocity of rotation of the LV around the axis of sensitivity $\omega_{0}$, and penetrating acoustic radiation (Figure 2).

Obviously, the components ${\left(\omega_{x}\right)}_{x_{1}}$ and ${\left(\omega_{z}\right)}_{x_{1}}$ do not affect the error of the gyroscope, as they coincide in direction with the axis of the figure. At the same time, kinematic perturbations ${\left(\omega_{x}\right)}_{z_{1}}$ and ${\left(\omega_{z}\right)}_{z_{1}}$ lead to additional measurement error (Figure 1) [1]:

(2) $$\omega_{1}^{a}\left(t\right)\approx \frac{2}{HR}(\omega_{x}\sin\beta +\omega_{z}\cos\beta )\left\{I_{\Pi }\left[\dot{V}\left(t\right)+\pi \dot{W}\left(t\right)\right]+m_{T}RL{\dot{W}}_{T}\left(t\right)\right\}\;=\frac{2}{HR}\left[\left(\dot{\theta }-\dot{\varphi }\sin\psi \right)\sin\beta +\left(\dot{\varphi }\cos\theta \cos\psi -\dot{\psi }\sin\theta \right)\cos\beta \right]\times \left\{I_{\Pi }\left[\dot{V}\left(t\right)+\pi \dot{W}\left(t\right)\right]+m_{T}RL{\dot{W}}_{T}\left(t\right)\right\}$$

The velocity $\omega_{1}^{a}\left(t\right)$ and angular acceleration ${\dot{\omega }}_{1}^{a}\left(t\right)$ vectors are directed along the output axis of the instrument.

From Equation (1) in this case we obtain:

(3) $$\frac{1}{2}R\omega_{0}{}^{2}\sin2\beta_{0}+H\omega_{0}\cos\beta_{0}+B{\dot{\omega }}_{1}^{a}+c\beta_{0}=0.$$

It follows from Expression (2) that

(4) $$\omega_{1}^{a}=\frac{2}{HR}\omega_{z}\cos\beta \left[I_{\Pi }\left(\dot{V}+\pi \dot{W}\right)+m_{T}R_{T}L{\dot{W}}_{T}\right]=\frac{2\omega_{z}}{HR}P_{0}i\omega_{a}\cos\beta \left[I_{\Pi }\left(\rho_{\tau }V+\rho_{r}\pi W\right)+m_{T}R_{T}L\rho_{T}W_{T}\right];$$

(5) $${\dot{\omega }}_{1}^{a}=\frac{2P_{0}i\omega_{a}}{HR}\left[I_{\Pi }\left(\rho_{\tau }V+\rho_{r}\pi W\right)+\rho_{T}m_{T}R_{T}LW_{T}\right]\times \left[{\dot{\omega }}_{z}\cos\beta +\omega_{z}\left(i\omega_{a}\cos\beta -\dot{\beta }\sin\beta \right)\right]$$

where $P_{0}$ is the pressure in the incident sound wave and $\omega_{a}$ is circular frequency of the acoustic wave.

Taking into account (5), Expression (3) takes the form:

(6) $$\frac{1}{2}R\omega_{0}^{2}\sin2\beta_{0}+H\omega_{0}\cos\beta_{0}++2BP_{0}\frac{i\omega_{a}}{HR}\left[I_{\Pi }\left(\rho_{\tau }V+k_{r}\pi W\right)+\rho_{T}m_{T}R_{T}LW_{T}\right]\omega_{0}i\omega_{a}\cos\beta_{0}+c\beta_{0}=0$$

Or this way:

(7) $$\frac{1}{2}R\omega_{0}^{2}\sin2\beta_{0}+\omega_{0}\left\{H\cos\beta_{0}-\right.2BP_{0}\frac{\omega_{a}^{2}\cos\beta_{0}}{HR}\times \left.\left[I_{\Pi }\left(\rho_{\tau }V+k_{r}\pi W\right)+\rho_{T}m_{T}R_{T}LW_{T}\right]\right\}+c\beta_{0}=0$$

From here, we find the relationship between the established value of the float rotation angle and the angular velocity of rotation around the axis of sensitivity:

(8) $$\omega_{0}=\frac{1}{R\sin2\beta_{0}}\left\{-H\cos\beta_{0}+2BP_{0}\frac{\omega_{a}^{2}\cos\beta_{0}}{HR}\left[I_{\Pi }\left(\rho_{\tau }V+\rho_{r}\pi W\right)+\rho_{T}m_{T}R_{T}LW_{T}\right]\right.\;+\sqrt{H^{2}\cos^{2}\beta_{0}+4B^{2}P_{0}^{2}\frac{\omega_{a}^{4}\cos^{2}\beta_{0}}{H^{2}R^{2}}{\left[I_{\Pi }\left(\rho_{\tau }V+\rho_{r}\pi W\right)+\rho_{T}m_{T}R_{T}LW_{T}\right]}^{2}}\;\left.\bar{-4HBP_{0}\frac{\omega_{a}^{2}\cos^{2}\beta_{0}}{HR}\left[I_{\Pi }\left(\rho_{\tau }V+\rho_{r}\pi W\right)+\rho_{T}m_{T}R_{T}LW_{T}\right]-2Rc\beta_{0}\sin2\beta_{0}}\right\}\;=\frac{1}{R\sin2\beta_{0}}\left\{-H\cos\beta_{0}+2BP_{0}\frac{\omega_{a}^{2}\cos\beta_{0}}{HR}\left[I_{\Pi }\left(\rho_{\tau }V+k_{r}\pi W\right)+\rho_{T}m_{T}R_{T}LW_{T}\right]\right.\;+H\cos\beta_{0}\sqrt{1+4B^{2}P_{0}^{2}\frac{\omega_{a}^{4}}{H^{4}R^{2}}{\left[I_{\Pi }\left(\rho_{\tau }V+k_{r}\pi W\right)+\rho_{T}m_{T}R_{T}LW_{T}\right]}^{2}}\;\left.\bar{-4BP_{0}\frac{\omega_{a}^{2}}{H^{2}R}\left[I_{n}\left(\rho_{\tau }V+\rho_{r}\pi W\right)+\rho_{T}m_{T}R_{T}LW_{T}\right]-2\frac{Rc\beta_{0}\sin2\beta_{0}}{H^{2}\cos\beta_{0}}}\right\}\;=\frac{1}{R\sin2\beta_{0}}\left\{\left\{-H\cos\beta_{0}+2BP_{0}\frac{\omega_{a}^{2}\cos\beta_{0}}{HR}\right.\right.\left[I_{\Pi }\left(\rho_{\tau }V+\rho_{r}\pi W\right)\right]\;+H\cos\beta_{0}\left\{1+2B^{2}P_{0}^{2}\frac{\omega_{a}^{4}}{H^{4}R^{2}}{\left[I_{\Pi }\left(\rho_{\tau }V+\rho_{r}\pi W\right)+\rho_{T}m_{T}R_{T}LW_{T}\right]}^{2}\right.\;\left.-\left.2BP_{0}\frac{\omega_{a}^{2}}{H^{2}R}\left[I_{\Pi }\left(\rho_{\tau }V+\rho_{r}\pi W\right)+\rho_{T}m_{T}R_{T}LW_{T}\right]-\frac{Rc\beta_{0}\sin2\beta_{0}}{H^{2}\cos^{2}\beta_{0}}\right\}\right\}\;=\frac{1}{R\sin2\beta_{0}}\left\{\left\{2BP_{0}\frac{\omega_{a}^{2}\cos\beta_{0}}{HR_{T}}\right.\right.\left[I_{\Pi }\left(\rho_{\tau }V+\rho_{r}\pi W\right)+\rho_{T}m_{T}R_{T}LW_{T}\right]\;+2B^{2}P_{{}_{0}}^{2}\frac{\omega_{a}^{4}\cos\beta_{0}}{H^{3}R^{2}}{\left[I_{\Pi }\left(\rho_{\tau }V+\rho_{r}\pi W\right)+\rho_{T}m_{T}R_{T}LW_{T}\right]}^{2}\;\left.\left.-2BP_{0}\frac{\omega_{a}^{2}\cos\beta_{0}}{HR}\left[I_{\Pi }\left(\rho_{\tau }V+\rho_{r}\pi W\right)+\rho_{T}m_{T}R_{T}LW_{T}\right]-\frac{Rc\beta_{0}\sin2\beta_{0}}{H\cos\beta_{0}}\right\}\right\}\;=\frac{1}{R\sin2\beta_{0}}\left\{\left\{2B^{2}P_{{}_{0}}^{2}\frac{\omega_{a}^{4}\cos\beta_{0}}{H^{2}R_{{}_{T}}^{2}}\right.\right.{\left\{I_{\Pi }\left[\rho_{\tau }V\left(t\right)+\rho_{r}\pi W\left(t\right)\right]+\rho_{T}m_{T}R_{T}LW_{T}\left(t\right)\right\}}^{2}\left.\left.-\frac{Rc\beta_{0}\sin2\beta_{0}}{H\cos\beta_{0}}\right\}\right\}$$

In the case when the penetrating acoustic effect is absent (for this in Formula (7), it is necessary to put $P_{0}=0$ and $\omega_{a}=0$), for small angles $\beta_{0}$ we obtain a known formula that determines the relationship between the established angle of the float rotation and a constant input value (fuselage circulation):

(9) $$\omega_{0}\approx -\frac{c}{H}\beta_{0}$$

Otherwise, in Formula (9) another term is added, that is:

(10) $$\omega_{0}\approx -\frac{c}{H}\beta_{0}+\frac{P_{0}^{2}}{\sin\beta_{0}}\frac{B^{2}}{R}\frac{\omega_{a}^{4}}{H^{3}R_{T}}\left\{I_{\Pi }\left[\rho_{\tau }V\left(z,\varphi ,\delta ,t\right)+\rho_{r}\pi W\left(z,\varphi ,\delta ,t\right)\right]+\right.\left.\rho_{T}m_{T}R_{T}LW_{T}\left(x,y,t\right)\right\}.$$

It is the second term that takes into account the influence of acoustic radiation on the device error at a constant value of the input quantity $\omega_{0}$. In Expression (10) $W_{T}\left(x,y,t\right)$ there is a law of bending motion of the float end under the action of penetrating acoustic radiation.

Consider the more general case where the angles $\omega_{x},\omega_{y}$, and $\omega_{z}$ are not equal to zero, but ψ and θ are small together with their derivatives, and the angular yaw rate is determined by the expression $\dot{\varphi }=\omega_{0}+\omega_{z}$.

Thus, neglecting the terms above the second order of smallness, we obtain:

(11) $$\omega_{x}=\dot{\theta }-\dot{\varphi }\sin\psi \approx \dot{\theta }-\omega_{0}\psi -\omega_{z}\psi ;\;\omega_{y}=\dot{\varphi }\sin\theta \cos\psi +\dot{\psi }\cos\theta \approx \omega_{0}\theta +\dot{\psi }+\omega_{z}\theta ;\;\omega_{z}=\dot{\varphi }\cos\theta \cos\psi -\dot{\psi }\sin\theta \approx \omega_{0}+\omega_{z}+\frac{1}{2}\omega_{0}\left(\theta^{2}+\psi^{2}\right)-\dot{\psi }\theta$$

Or in this form:

(12) $$\omega_{x}=\omega_{1x}+\omega_{2x};\;\omega_{y}=\omega_{1y}+\omega_{2y};\;\omega_{z}=\omega_{0}+\omega_{1z}+\omega_{2z}$$

where $\omega_{1x}=\dot{\theta }-\omega_{0}\psi ;$ $\omega_{2x}=-\omega_{z}\psi ;$ $\omega_{1y}=\dot{\psi }+\omega_{0}\theta ;$ $\omega_{2y}=\omega_{z}\theta ;$ $\omega_{1z}=\omega_{z};$ $\omega_{2z}=\frac{1}{2}\omega_{0}(\theta^{2}+\psi^{2})-\dot{\psi }\theta ;$ $\omega_{ij}(i=1,2;j=x,y,z)$—respectively, the components of the angular velocity $\omega_{j}$ of the first (i=1) and second (i=2) order of smallness.

Before substituting (12) into Equation (1), we perform here the decomposition of the function β and trigonometric functions into series in the neighborhood of the value $\beta_{0}$ satisfying (3):

(13) $$\beta =\beta_{0}+\beta_{1}+\beta_{2}+\ldots ;\;\sin\beta =\sin\beta_{0}+\beta_{1}\cos\beta_{0}-\frac{1}{2}\beta_{{}^{1}}^{2}\sin\beta_{0}+\beta_{2}\cos\beta_{0}+\ldots ;\;\cos\beta =\cos\beta_{0}-\beta_{1}\sin\beta_{0}-\frac{1}{2}\beta_{{}^{1}}^{2}\cos\beta_{0}-\beta_{2}\sin\beta_{0}+\ldots ;\;\sin2\beta =\sin2\beta_{0}+2\beta_{1}\cos2\beta_{0}-2\beta_{{}^{1}}^{2}\sin2\beta_{0}+2\beta_{2}\cos2\beta_{0}+\ldots ;\;\cos2\beta =\cos2\beta_{0}-2\beta_{1}\sin2\beta_{0}-2\beta_{{}^{1}}^{2}\cos2\beta_{0}-2\beta_{2}\sin2\beta_{0}+\ldots$$

Equation (1) after substituting relations (13) in (1) gives:

(14) $$\begin{array}{c} B\left({\ddot{\beta }}_{1}++{\ddot{\beta }}_{2}\right)+R\left[\frac{1}{2}\omega_{0}^{2}\sin2\beta_{0}+\omega_{0}^{2}\beta_{1}\cos2\beta_{0}+\omega_{0}\omega_{1z}\sin2\beta_{0}+2\omega_{0}\omega_{1z}\beta_{1}\cos2\beta_{0}\right.\\ -2\omega_{0}^{2}\beta_{1}^{2}\sin2\beta_{0}+\frac{1}{2}\left(\omega_{1z}^{2}+2\omega_{0}\omega_{2z}+2\omega_{0}\omega_{2y}Q_{1}\cos\beta_{0}+\omega_{{}_{1y}}^{2}Q_{1}^{2}\cos^{2}\beta_{0}\right)\\ +\omega_{0}^{2}\beta_{2}\cos2\beta_{0}-\frac{1}{2}\omega_{1x}^{2}\sin2\beta_{0}-\omega_{0}\omega_{1x}\cos2\beta_{0}+\omega_{0}\omega_{1y}Q_{1}\sin2\beta_{0}\cos\beta_{0}\\ +2\omega_{0}\omega_{1x}\beta_{1}\sin2\beta_{0}+2\omega_{0}\omega_{1y}\beta_{1}Q_{1}(\cos2\beta_{0}\cos\beta_{0}-\frac{1}{2}\sin2\beta_{0}\sin\beta_{0})\\ -\left(\omega_{0}\omega_{2x}+\omega_{1x}\omega_{1z}+\omega_{1x}\omega_{1z}\right.Q_{1}\cos\beta_{0}-\omega_{1y}\omega_{1z}Q_{1}tg2\beta_{0}\cos\left.\beta_{0}\right)]\\ +H(\omega_{1x}\sin\beta_{0}+\omega_{0}\cos\beta_{0}+\omega_{1z}\cos\beta_{0}+\omega_{0}\beta_{1}\sin\beta_{0}+\omega_{2x}\sin\beta_{0}\\ +\omega_{2z}\cos\beta_{0}+\omega_{1x}\beta_{1}\cos\beta_{0}-\omega_{1z}\beta_{1}\sin\beta_{0}-\frac{1}{2}\omega_{0}\beta_{{}_{1}}^{2}\cos\beta_{0}-\omega_{0}\beta_{2}\sin\beta_{0}\\ \left.+Q_{1}\omega_{1y}\cos^{2}\beta_{0}-\omega_{1y}\beta_{1}Q_{1}\sin2\beta_{0}+\omega_{2y}Q_{1}\cos^{2}\beta_{0}\right)\\ +B\left[{\dot{\omega }}_{1y}\right.+{\dot{\omega }}_{2y}+Q\omega_{0}i\omega_{a}\cos\beta_{0}+\left({\dot{\omega }}_{1z}-\omega_{1z}i\omega_{a}\right)Q\cos\beta_{0}\\ -\omega_{0}\left({\dot{\beta }}_{1}+\beta_{1}i\omega_{a}\right)Q\sin\beta_{0}-\omega_{1z}Q\sin\beta_{0}\left(i\omega_{a}\beta_{1}+2{\dot{\beta }}_{1}\right)+Q\cos\beta_{0}\left({\dot{\omega }}_{2z}+\omega_{2z}i\omega_{a}\right)\\ -Q\omega_{0}\cos\beta_{0}\left(\frac{1}{2}\beta_{1}^{2}i\omega_{a}+{\dot{\beta }}_{1}\beta_{1}\right)-Q\omega_{0}\sin\beta_{0}\left.\left(\beta_{2}i\omega_{a}+{\dot{\beta }}_{2}\right)\right]+b\left({\dot{\beta }}_{1}+{\dot{\beta }}_{2}\right)+c\left(\beta_{1}+\beta_{2}\right)=0 \end{array}$$

where $Q_{1}=\frac{4\rho_{r}}{HR}I_{\Pi }\dot{W}\left(t\right)=\frac{4P_{0}}{HR}i\omega_{a}I_{\Pi }\rho_{r}$; $Q=\frac{2P_{0}}{HR}i\omega_{a}\left[I_{\Pi }\left(\rho_{\tau }+\rho_{r}\pi \right)+\rho_{T}m_{T}R_{T}L\right]$.

The equation for determining the first approximation will look like:

(15) $$B{\ddot{\beta }}_{1}+\left(b-\omega_{0}Q\sin\beta_{0}\right){\dot{\beta }}_{1}+\left(c+\omega_{0}r_{1}-i\omega_{a}\omega_{0}Q\sin\beta_{0}\right)\beta_{1}=r_{1}\omega_{1x}\;-\left(q_{1}-i\omega_{a}Q\cos\beta_{0}\right)\omega_{1z}-B{\dot{\omega }}_{1y}+q_{1}Q_{1}\cos\beta_{0}\omega_{1y}+Q\cos\beta_{0}{\dot{\omega }}_{1z}$$

where $r_{1}=R\omega_{0}\cos2\beta_{0}-H\sin\beta_{0}$; $q_{1}=R\omega_{0}\sin2\beta_{0}+H\cos\beta_{0}$; $b^{a}=\omega_{0}Q\sin\beta_{0}$; $r_{1}^{a}=i\omega_{a}Q\sin\beta_{0}$; $q_{1}^{a}=i\omega_{a}Q\cos\beta_{0}$.

Defining ψ,θ, and $\omega_{z}$ as functions of time, it is possible to find $\omega_{1x},\omega_{1y},\omega_{1z},{\dot{\omega }}_{1y}$ and ${\dot{\omega }}_{1z}$ from relations (11), and having substituted in the Equation (15), it is possible to calculate $\beta_{1}$.

The equation of the second approximation is obtained from (14) by equating in its left and right parts the members of the second order of smallness. It turns out that the left-hand side of the equation has the same form as (15). Only the right parts differ:

(16) $$\begin{array}{l} B{\ddot{\beta }}_{2}+(b-\omega_{0}Q\sin\beta_{0}){\dot{\beta }}_{2}+\left(c+\omega_{0}r_{1}-i\omega_{a}\omega_{0}Q\sin\beta_{0}\right)\beta_{2}\\ \;\;\;\;\;\;=r_{1}\omega_{2x}+\left(2R\omega_{0}+H\cos\beta_{0}\right)Q_{1}\cos\beta_{0}\omega_{2y}-\left(q_{1}+q_{1}^{'}+2i\omega_{a}Q\cos\beta_{0}\right)\omega_{2z}\\ \;\;\;\;\;\;+r_{1}^{'}\beta_{1}\omega_{1x}+\left[R\omega_{0}\cos2\beta_{0}\cos\beta_{0}\left(2-tq2\beta_{0}tq\beta_{0}\right)-H\sin2\beta_{0}\right]Q_{1}\beta_{1}\omega_{1y}-q_{1}^{'}\beta_{1}\omega_{1z}\\ \;\;\;\;\;\;+Q\cos\beta_{0}\omega_{0}\beta_{1}{\dot{\beta }}_{1}-2Q\sin\beta_{0}{\dot{\beta }}_{1}\omega_{1z}-\frac{1}{2}\omega_{0}\left(q_{1}^{\prime \prime }+i\omega_{a}Q\cos\beta_{0}\right)\beta_{1}^{2}\\ \;\;\;\;\;\;+\frac{1}{2}R\left[\left(\omega_{1x}^{2}-\omega_{1z}^{2}\right)\sin2\beta_{0}+2\omega_{1y}^{2}Q_{1}^{2}\cos^{2}\beta_{0}+2\omega_{1x}\omega_{1z}\cos2\beta_{0}\right.\\ \;\;\;\;\;\;+2\omega_{1x}\omega_{1y}Q_{1}\cos\beta_{0}--2\omega_{1y}\omega_{1z}tg2\beta_{0}\cos\beta_{0}]-B{\dot{\omega }}_{2y}+{\dot{\omega }}_{2z}Q\cos\beta_{0} \end{array}$$

where $q_{1}^{\prime }=2R\omega_{0}\cos2\beta_{0}-H\sin\beta_{0}$; $q_{1}^{\prime \prime }=-2R\omega_{0}\sin2\beta_{0}+H\cos\beta_{0}$.

We introduce such notations:

$$\begin{array}{l} \omega_{0}Q\sin\beta_{0}=b^{a};\;i\omega_{a}Q\sin\beta_{0}=r_{1}^{a};\;i\omega_{a}Q\cos\beta_{0}=q_{1}^{a};\;Q_{1}\cos\beta_{0}=\lambda ;\\ Q\cos\beta_{0}=\mu^{a};\;\frac{c}{B}=k^{2};\;\frac{r_{1}}{B}=r;\;\frac{q_{1}}{B}=q;\;\frac{q_{1}^{a}}{B}=q^{a};\;\frac{r_{1}^{a}}{B}=r^{a};\\ n^{2}=k^{2}+\omega_{0}\left(r-r^{a}\right);\;\frac{\mu^{a}}{B}=\mu ;\;\frac{b}{B}=2h;\;\frac{b^{a}}{B}=2h^{a}. \end{array}$$

Then, Equation (15) takes the form:

(17) $$B{\ddot{\beta }}_{1}+\left(b-b^{a}\right){\dot{\beta }}_{1}+\left(c+\omega_{0}r_{1}-\omega_{0}r_{1}^{a}\right)\beta_{1}\;=r_{1}\omega_{1x}-\left(q_{1}-q_{1}^{a}\right)\omega_{1z}+q_{1}\lambda \omega_{1y}+\mu^{a}{\dot{\omega }}_{1z}-B{\dot{\omega }}_{1y}$$

After division by the value of B, we obtain the final:

(18) $${\ddot{\beta }}_{1}+\left(2h-2h^{a}\right){\dot{\beta }}_{1}+n^{2}\beta_{1}=r\omega_{1x}-\left(q-q^{a}\right)\omega_{1z}+q\lambda \omega_{1y}+\mu {\dot{\omega }}_{1z}-{\dot{\omega }}_{1y}$$

Similarly, for Equation (16) of the second approximation:

(19) $${\ddot{\beta }}_{2}+\left(2h-2h^{a}\right){\dot{\beta }}_{2}+n^{2}\beta_{2}=r\omega_{2x}+S\lambda \omega_{2y}-\left(q+q^{\prime }+q^{a}\right)\omega_{2z}+\beta_{1}r^{\prime }\omega_{1x}\;+l^{\prime }\beta_{1}\omega_{1y}-q^{\prime }\beta_{1}\omega_{1z}+\mu \omega_{0}\beta_{1}{\dot{\beta }}_{1}+2\mu^{\prime }{\dot{\beta }}_{1}\omega_{1z}\;\;\;\;\;-\frac{1}{2}\omega_{0}\left(q^{\prime \prime }+q^{a}\right)\beta_{1}^{2}+\frac{a}{2}\left[\left(\omega_{1x}^{2}-\omega_{1z}^{2}\right)\sin2\beta_{0}\right.+2\omega_{1y}^{2}\lambda^{2}+2\omega_{1x}\omega_{1z}\cos2\beta_{0}+2\omega_{1x}\omega_{1y}\lambda \;\;\;\;\;\;\;\;\;-2\omega_{1y}\omega_{1z}tg2\beta_{0}\cdot \cos\beta_{0}]-{\dot{\omega }}_{2y}+\mu {\dot{\omega }}_{2z}.\;\;\;\;\;\;\;\;\;\;\;\;\;\;\;\;\;\;\;\;\;\;\;\;\;\;\;\;\;\;\;\;\;\;\;\;\;\;\;\;\;\;\;\;\;\;\;\;\;\;\;\;\;\;\;\;\;\;\;\;\;\;\;\;\;\;\;\;\;\;\;\;\;\;\;\;\;\;\;$$

Or this way:

(20) $${\ddot{\beta }}_{2}+\left(2h-2h^{a}\right){\dot{\beta }}_{2}+n^{2}\beta_{2}=r\omega_{2x}+S\lambda \omega_{2y}-\left(q+q^{\prime }+q^{a}\right)\omega_{2z}\;+\beta_{1}\left(r^{\prime }\omega_{1x}+l^{\prime }\omega_{1y}-q^{\prime }\omega_{1z}\right)+{\dot{\beta }}_{1}\left(\mu \omega_{0}\beta_{1}+2\mu^{\prime }\omega_{1z}\right)-\frac{1}{2}\omega_{0}\left(q^{\prime \prime }+q^{a}\right)\beta_{1}^{2}\;+\frac{a}{2}\left[\left(\omega_{1x}^{2}-\omega_{1z}^{2}\right)\sin2\beta_{0}+2\lambda^{2}\omega_{1y}^{2}+2\omega_{1x}\omega_{1z}\cos2\beta_{0}\right.\;+2\lambda \omega_{1x}\omega_{1y}-2tg2\beta_{0}\omega_{1y}\omega_{1z}\cdot \cos\beta_{0}]-{\dot{\omega }}_{2y}+\mu {\dot{\omega }}_{2z}$$

where $(2R\omega_{0}+H\cos\beta_{0})=S^{a}$; $\frac{S^{a}}{B}=S$; $\frac{{q^{\prime }}_{1}}{B}=q^{\prime }$; $\frac{{r^{\prime }}_{1}}{B}=r^{\prime }$; $\left[R\omega_{0}\cos2\beta_{0}\cos\beta_{0}\left(2-tg2\beta_{0}tg\beta_{0}\right)-H\sin2\beta_{0}\right]Q_{1}=l^{a}$; $\frac{l^{a}}{B}=l^{\prime };$ $\frac{{q^{\prime \prime }}_{1}}{B}=q^{\prime \prime }$; $\frac{{\left(\mu^{a}\right)}^{\prime }}{B}=\mu^{\prime };$ $\frac{{q^{\prime \prime }}_{1}}{B}=q^{\prime \prime }$; $\frac{R}{B}=a$.

### 2.1. The First Approximation

The general solution of the equation of the first approximation (18) can be represented by the sum of the general solution of the homogeneous equation and the partial solution of the inhomogeneous equation, i.e.,

(21) $$\beta_{1}=C\exp\left[-\left(h-h^{a}\right)\sin(\sqrt{n^{2}{\left(h-h^{a}\right)}^{2}}t+\varepsilon )+{\tilde{\beta }}_{1}\right]$$

It is obvious that over time, the first term here decreases t→∞ and tends to zero. Therefore, the established value will be determined by a particular solution ${\tilde{\beta }}_{1}$.

Let us analyze the reaction of the float gyroscope to the harmonic oscillations of the base. Suppose first that the right-hand side of Equation (18) is a harmonic function of, for example, the form that is

(22) $$f\left(t\right)=\rho \sin\left(\nu t+\delta \right)\;{\ddot{\beta }}_{1}+2\left(h-h^{a}\right){\dot{\beta }}_{1}+n^{2}\beta_{1}=\rho \sin\left(\nu t+\delta \right)$$

The steady movement in this case will also be periodic. It is determined from the solution of Equation (22):

(23) $$\beta_{1}={\left\{\;{\left[n^{2}-\nu^{2}\right]}^{2}+4{\left(h-h^{a}\right)}^{2}\nu^{2}\right\}}^{-\frac{1}{2}}\rho \sin\left(\nu t+\delta -\varepsilon \right)$$

where

(24) $$\varepsilon =arctg\frac{2\left(h-h^{a}\right)}{n^{2}-\nu^{2}},\;\mathrm{if}\;n>v;\;\varepsilon =\pi +arctg\frac{2\left(h-h^{a}\right)\nu }{n^{2}-\nu^{2}},\;\mathrm{if}\;n<v;$$

Note that if in the right-hand side of Equation (22) the periodic function is cosine, then

$$\beta_{1}={\left\{\;{\left[n^{2}-\nu^{2}\right]}^{2}-4{\left(h-h^{a}\right)}^{2}\nu^{2}\right\}}^{-\frac{1}{2}}\rho \cos\left(\nu t+\delta -\varepsilon \right)$$

Let the angular oscillations of the LV body occur according to the harmonic law, that is

(25) $$\theta =\rho_{\theta }\sin\left(\nu t+\delta_{\theta }\right);\;\psi =\rho_{\psi }\sin\left(\nu t+\delta_{\psi }\right);\;\omega_{1z}=\nu \rho_{\varphi }\sin\left(\nu t+\delta_{\varphi }\right),$$

and the acoustic vibration of the float surface also occurs according to the harmonic law—

(26) $$V\left(t\right)=\rho_{\tau }\cos\left(\omega_{a}t+\delta_{V}\right);\;W\left(t\right)=\rho_{r}\cos\left(\omega_{a}t+\delta_{W}\right);\;W_{T}\left(t\right)=\rho_{T}\cos\left(\omega_{a}t+\delta_{W_{T}}\right).$$

In acoustics, only the cosine component is often used and the imaginary sine component is omitted.

The right-hand side of Equation (28) in this case will take the form:

(27) $$r\omega_{1x}-\left(q-q^{a}\right)\omega_{1z}+q\lambda \omega_{1y}+\mu {\dot{\omega }}_{1z}-{\dot{\omega }}_{1y}=\left(r-\omega_{0}\right)\nu \rho_{\theta }\cos\left(\nu t+\delta_{\theta }\right)\;-\left(r\omega_{0}-\nu^{2}\right)\rho_{\psi }\sin\left(\nu t+\delta_{\psi }\right)-q\nu \rho_{\phi }\cos\left(\nu t+\delta_{\phi }\right)-\frac{2P_{0}}{BHR}\omega_{a}^{2}\cos\beta_{0}\;\times [I_{\Pi }\left(\rho_{\tau }\cos\left(\omega_{a}t+\delta_{V}\right)\right)+\pi \rho_{r}\cos\left(\omega_{a}t+\delta_{W}\right)+m_{T}R_{T}L\rho_{T}\cos\left(\omega_{a}t+\delta_{W_{T}}\right)\;\times \nu \rho_{\phi }\cos\left(\nu t+\delta_{\phi }\right)\;+\frac{4P_{0}}{HBR}\left(R\omega_{0}\sin2\beta_{0}+H\cos\beta_{0}\right)i\omega_{a}\cos^{2}\beta_{0}I_{\Pi }\rho_{r}\cos\left(\omega_{a}t+\delta_{W}\right)\;\times \left[\nu \rho_{\psi }\cos\left(\nu t+\delta_{\psi }\right)+\omega_{0}\rho_{\theta }\sin\left(\nu t+\delta_{\theta }\right)\right]\;+\frac{2P_{0}i\omega_{a}\cos\beta_{0}}{HBR}\left[I_{\Pi }\left(\rho_{\tau }\cos\left(\omega_{a}t+\delta_{V}\right)\right.\right.\;+\rho_{r}\pi \cos\left(\omega_{a}t+\delta_{W}\right)+\rho_{T}m_{T}R_{T}L\cos\left(\omega_{a}t+\delta_{W_{T}}\right)]\nu^{2}\rho_{\phi }\sin\left(\nu t+\delta_{\phi }\right)\;+\nu^{2}\rho_{\psi }\sin\left(\nu t+\delta_{\psi }\right)-\nu \omega_{0}\rho_{\theta }\cos\left(\nu t+\delta_{\theta }\right)\;=\left(r-\omega_{0}\right)\nu \rho_{\theta }\cos\left(\nu t+\delta_{\theta }\right)-\left(r\omega_{0}-\nu^{2}\right)\rho_{\psi }\sin\left(\nu t+\delta_{\psi }\right)-q\nu \rho_{\phi }\cos\left(\nu t+\delta_{\phi }\right)\;-\frac{P_{0}\omega_{a}^{2}\cos\beta_{0}}{BHR}\left\{I_{\Pi }\right.\nu \rho_{\tau }\rho_{\phi }\cos\left[\left(\omega_{a}-\nu \right)t+\delta_{V}-\delta_{\phi }\right]\;+I_{\Pi }\nu \rho_{\tau }\rho_{\phi }\cos[\left(\omega_{a}+\nu \right)t+\delta_{V}+\left.\delta_{\phi }\right]+I_{\Pi }\pi \nu \rho_{r}\rho_{\phi }\cos[\left(\omega_{a}-\nu \right)t+\delta_{W}-\delta_{\phi }]\;+I_{\Pi }\pi \nu \rho_{r}\rho_{\phi }\cos[\left(\omega_{a}+\nu \right)t+\delta_{W}+\delta_{\phi }]+m_{T}R_{T}L\nu \rho_{T}\rho_{\phi }\cos[\left(\omega_{a}-\nu \right)t+\delta_{W_{T}}-\delta_{\phi }]\;\left.+m_{T}R_{T}L\nu \rho_{T}\rho_{\phi }\cos[\left(\omega_{a}+\nu \right)t+\delta_{W_{T}}+\delta_{\phi }]\right\}\;+\frac{2P_{0}i\omega_{a}\cos^{2}\beta_{0}I_{\Pi }\left(R\omega_{0}\sin2\beta_{0}+H\cos\beta_{0}\right)}{HBR}\left\{\nu \rho_{r}\rho_{\psi }\cos\left[\left(\omega_{a}-\nu \right)t+\delta_{W}-\delta_{\psi }\right]\right.\;+\nu \rho_{r}\rho_{\psi }\cos[\left(\omega_{a}+\nu \right)t+\delta_{W}+\delta_{\psi }]+\omega_{0}\rho_{r}\rho_{\theta }\sin\left[\left(\omega_{a}-\nu \right)t+\delta_{W}-\delta_{\theta }\right]\;\left.+\omega_{0}\rho_{r}\rho_{\theta }\sin\left[\left(\omega_{a}+\nu \right)t+\delta_{W}+\delta_{\theta }\right]\right\}+\frac{P_{0}i\omega_{a}\cos\beta_{0}}{HBR}\left\{I_{\Pi }\nu^{2}\rho_{\tau }\rho_{\phi }\sin\left[\left(\omega_{a}-\nu \right)t+\delta_{V}-\delta_{\phi }\right]\right.\;+I_{\Pi }\nu^{2}\rho_{\tau }\rho_{\phi }\sin[\left(\omega_{a}+\nu \right)t+\delta_{V}+\delta_{\phi }]+\nu^{2}m_{T}R_{T}L\rho_{T}\rho_{\phi }\sin[\left(\omega_{a}-\nu \right)t+\delta_{W_{T}}-\delta_{\phi }]\;+\left.\nu^{2}m_{T}R_{T}L\rho_{T}\rho_{\phi }\sin[\left(\omega_{a}+\nu \right)t+\delta_{W_{T}}+\delta_{\phi }]\right\}+\nu^{2}\rho_{\psi }\sin\left(\nu t+\delta_{\psi }\right)\;-\nu \omega_{0}\rho_{\theta }\cos\left(\nu t+\delta_{\theta }\right).$$

Now we can use Solution (23) and determine $\beta_{1}$ for the case when the right-hand side of Equation (22) is represented in the form (27).

As follows from Expression (27), the right-hand side of the equation of the first approximation (18) will contain terms representing periodic functions of time. Moreover, some terms depend only on the frequency ν of angular motion of the launch vehicle, others, on the sum (or difference) of the frequency of acoustic radiation $\omega_{a}$ and kinematic perturbation ν. The first, of course, will lead to forced oscillations of the moving part of the device relative to the equilibrium position $\beta =\beta_{0}$ with frequency ν. The second, the total frequency ($\omega_{a}+\nu$), will also cause the float to oscillate relative to the output axis, but due to the combined action of two perturbing factors—the penetrating acoustic radiation and the angular motion of the LV housing. As follows from expression (27) when $\omega_{a}=0$ that is, in the absence of sound impact, the forced oscillations of the moving part will occur only due to the influence of the angular motion of the rocket.

Special attention should be paid to the analysis of the difference frequency components $\left(\omega_{a}-\nu \right)$:

(28) $$-\frac{P_{0}\omega_{a}^{2}\cos\beta_{0}}{HBR}\left\{I_{\Pi }\right.\nu \rho_{\tau }\delta_{\phi }\cos\left[\left(\omega_{a}-\nu \right)t+\delta_{V}-\delta_{\phi }\right]\;+\left.\pi \nu \rho_{r}\rho_{\phi }\cos[\left(\omega_{a}-\nu \right)t+\delta_{W}-\delta_{\phi }]+\nu m_{T}R_{T}L\rho_{T}\rho_{\phi }\cos[\left(\omega_{a}-\nu \right)t+\delta_{W_{T}}-\delta_{\phi }]\right\}\;+\frac{2P_{0}i\omega_{a}\cos^{2}\beta_{0}I_{\Pi }\left(R\omega_{0}\sin2\beta_{0}+H\cos\beta_{0}\right)}{HBR}\left\{\nu \rho_{r}\rho_{\psi }\cos\left[\left(\omega_{a}-\nu \right)t+\delta_{W}-\delta_{\psi }\right]\right.\;+\left.\omega_{0}\rho_{r}\rho_{\psi }\sin[\left(\omega_{a}-\nu \right)t+\delta_{W}-\delta_{\psi }]\right\}\;+\frac{P_{0}i\omega_{a}\cos\beta_{0}}{HBR}\left\{\nu^{2}I_{\Pi }\rho_{\tau }\rho_{\phi }\sin\left[\left(\omega_{a}-\nu \right)t+\delta_{V}-\delta_{\phi }\right]\right.\;+\left.\nu^{2}m_{T}R_{T}L\rho_{T}\rho_{\phi }\sin[\left(\omega_{a}-\nu \right)t+\delta_{W_{T}}-\delta_{\phi }]\right\}.$$

The case of frequency matching $\omega_{a}=\nu$ is interesting. As follows from (27), the summands containing $\sin(\omega_{a}-\nu )t,$ disappear, and the summands containing $\cos(\omega_{a}-\nu )t,$ turn into one, and Expression (27) takes the form:

(29) $$-\frac{P_{0}\omega_{a}^{2}\cos\beta_{0}}{HBR}\nu \left\{I_{\Pi }\right.\rho_{\tau }\delta_{\phi }\cos\left(\delta_{V}-\delta_{\phi }\right)\;+\left.\pi \rho_{r}\rho_{\phi }\cos\left(\delta_{W}-\delta_{\phi }\right)+m_{T}R_{T}L\rho_{T}\rho_{\phi }\cos\left(\delta_{W_{T}}-\delta_{\phi }\right)\right\}\;+\frac{2P_{0}i\omega_{a}\cos^{2}\beta_{0}I_{\Pi }\left(R\omega_{0}\sin2\beta_{0}+H\cos\beta_{0}\right)}{HBR}\left\{\nu \rho_{r}\rho_{\psi }\cos\left(\delta_{W}-\delta_{\psi }\right)\right.\;+\left.\omega_{0}\rho_{r}\rho_{\psi }\sin\left(\delta_{W}-\delta_{\psi }\right)\right\}+\frac{P_{0}i\omega_{a}\cos\beta_{0}}{HBR}\nu^{2}\left\{I_{\Pi }\rho_{\tau }\rho_{\phi }\sin\left(\delta_{V}-\delta_{\phi }\right)\right.\;+\left.m_{T}R_{T}L\rho_{T}\rho_{\phi }\sin\left(\delta_{W_{T}}-\delta_{\phi }\right)\right\}=-\frac{P_{0}\omega_{a}^{2}\cos\beta_{0}}{HBR}\nu \rho_{\phi }\;\times \left[I_{\Pi }\rho_{\tau }\cos\left(\delta_{V}-\delta_{\phi }\right)+\pi \rho_{r}\cos\left(\delta_{W}-\delta_{\phi }\right)+m_{T}R_{T}L\rho_{T}\cos\left(\delta_{W_{T}}-\delta_{\phi }\right)\right]\;+\frac{2P_{0}i\omega_{a}\cos^{2}\beta_{0}I_{\Pi }\left(R\omega_{0}\sin2\beta_{0}+H\cos\beta_{0}\right)}{HBR}\rho_{r}\rho_{\psi }\left[\nu \cos\left(\delta_{W}-\delta_{\psi }\right)\right.\;+\left.\omega_{0}\cos\left(\delta_{W}-\delta_{\psi }\right)\right]+\frac{P_{0}i\omega_{a}\cos\beta_{0}}{HBR}\nu^{2}\rho_{\phi }\left[I_{\Pi }\rho_{\tau }\sin\left(\delta_{V}-\delta_{\phi }\right)\right.\;+m_{T}R_{T}L\rho_{T}\sin\left(\delta_{W_{T}}-\delta_{\phi }\right)].$$

As can be seen, acoustic radiation will be a kind of filter, emphasizing the frequency band of kinematic perturbation. The presence of constants in the right part of the equation will lead to the detection in the first approximation of the systematic components in the output signal of the device.

Now we can use Solution (23) and determine $\beta_{1}$ for the case when the right-hand side of Equation (22) is represented as (27):

(30) $$\beta_{1}={\left[{\left(n^{2}-\nu^{2}\right)}^{2}+4{\left(h-h^{a}\right)}^{2}\nu^{2}\right]}^{-\frac{1}{2}}\;\times \left\{\left\{\left(r-\omega_{0}\right)\nu \rho_{\theta }\cos\left(\nu t+\delta_{\theta }-\varepsilon \right)-\left(r\omega_{0}-\nu^{2}\right)\rho_{\psi }\sin\left(\nu t+\delta_{\psi }-\varepsilon \right)\right.\right.\;-q\nu \rho_{\phi }\cos\left(\nu t+\delta_{\phi }-\varepsilon \right)-\frac{P_{0}\omega_{a}^{2}\cos\beta_{0}}{HBR}\nu \rho_{\phi }\left\{I_{\Pi }\right.\rho_{\tau }\cos\left[\left(\omega_{a}-\nu \right)t+\delta_{V}-\delta_{\phi }-\varepsilon \right]\;+I_{\Pi }\rho_{\tau }\cos[\left(\omega_{a}+\nu \right)t+\delta_{V}+\delta_{\phi }-\varepsilon ]+\pi \rho_{r}I_{\Pi }\cos[\left(\omega_{a}-\nu \right)t+\delta_{W}-\delta_{\phi }-\varepsilon ]\;+\pi \rho_{r}I_{\Pi }\cos[\left(\omega_{a}+\nu \right)t+\delta_{W}+\delta_{\phi }-\varepsilon ]+m_{T}R_{T}L\rho_{T}\cos[\left(\omega_{a}-\nu \right)t+\delta_{W_{T}}-\delta_{\phi }-\varepsilon ]\;+\left.m_{T}R_{T}L\rho_{T}\cos[\left(\omega_{a}+\nu \right)t+\delta_{W_{T}}+\delta_{\phi }-\varepsilon ]\right\}\;+\frac{2P_{0}i\omega_{a}\cos^{2}\beta_{0}I_{\Pi }\left(R\omega_{0}\sin2\beta_{0}+H\cos\beta_{0}\right)}{HBR}\left\{\nu \rho_{r}\rho_{\psi }\cos\left[\left(\omega_{a}-\nu \right)t+\delta_{W}-\delta_{\psi }-\varepsilon \right]\right.\;+\nu \rho_{r}\rho_{\psi }\cos[\left(\omega_{a}+\nu \right)t+\delta_{W}+\delta_{\psi }-\varepsilon ]+\omega_{0}\rho_{r}\rho_{\theta }\sin[\left(\omega_{a}-\nu \right)t+\delta_{W}-\delta_{\theta }-\varepsilon ]\;\left.+\omega_{0}\rho_{r}\rho_{\theta }\sin\left[\left(\omega_{a}+\nu \right)t+\delta_{W}+\delta_{\theta }-\varepsilon \right]\right\}\;+\frac{P_{0}i\omega_{a}\cos\beta_{0}}{HBR}\nu^{2}\rho_{\phi }\left\{I_{\Pi }\rho_{\tau }\sin\left[\left(\omega_{a}-\nu \right)t+\delta_{V}-\delta_{\phi }-\varepsilon \right]\right.\;+I_{\Pi }\rho_{\tau }\sin[\left(\omega_{a}+\nu \right)t+\delta_{V}+\delta_{\phi }-\varepsilon ]+m_{T}R_{T}L\rho_{T}\sin[\left(\omega_{a}-\nu \right)t+\delta_{W_{T}}-\delta_{\phi }-\varepsilon ]\;\left.\left.+\left.m_{T}R_{T}L\rho_{T}\sin\left[\left(\omega_{a}+\nu \right)t+\delta_{W_{T}}+\delta_{\phi }-\varepsilon \right]\right\}\right\}\right\}.$$

The first three terms in the right-hand side of Expression (30) describe the effect of only the angular motion of the rocket body on the output signal, the others, the combined effect of acoustic and kinematic perturbations.

For asynchronous oscillations, when

(31) $$\theta =\rho_{\theta }\sin\left(\nu_{1}t+\delta_{\theta }\right);\;\psi =\rho_{\psi }\sin\left(\nu_{2}t+\delta_{\psi }\right);\;\omega_{1z}=\nu_{3}\rho_{\phi }\cos\left(\nu_{3}t+\delta_{\phi }\right);\;V\left(t\right)=\rho_{\tau }\cos\left(\omega_{a}t+\delta_{V}\right);\;W\left(t\right)=\rho_{r}\cos\left(\omega_{a}t+\delta_{W}\right);\;W_{T}\left(t\right)=\rho_{T}\cos\left(\omega_{a}t+\delta_{W_{T}}\right).$$

Expression (30) will change:

(32) $$\beta_{1}=\left(r-\omega_{0}\right)D\left(\nu_{1}\right)\nu_{1}\rho_{\theta }\cos\left(\nu_{1}t+\delta_{\theta }-\varepsilon_{1}\right)+\left(r\omega_{0}-\nu_{2}^{2}\right)\rho_{\psi }\sin\left(\nu_{2}t+\delta_{\psi }-\varepsilon_{2}\right)D\left(\nu_{2}\right)\;-qD\left(\nu_{3}\right)\nu_{3}\rho_{\phi }\cos\left(\nu_{3}t+\delta_{\phi }-\varepsilon_{3}\right)-\omega_{0}D\left(\nu_{2}\right)\nu_{2}^{2}\rho_{\psi }\sin\left(\nu_{2}t+\delta_{\psi }-\varepsilon_{2}\right)\;-\omega_{0}D\left(\nu_{1}\right)\nu_{1}\rho_{\theta }\sin\left(\nu_{1}t+\delta_{\theta }-\varepsilon_{1}\right)-\frac{P_{0}\omega_{a}^{2}\cos\beta_{0}}{HBR}D\left(\nu_{3}\right)\nu_{3}\rho_{\phi }\;\times \left\{I_{\Pi }\rho_{\tau }\cos[\left(\omega_{a}-\nu_{3}\right)t+\delta_{V}-\delta_{\phi }-\varepsilon_{3}]+I_{\Pi }\rho_{\tau }\cos[\left(\omega_{a}+\nu_{3}\right)t+\delta_{V}+\delta_{\phi }-\varepsilon_{3}]\right.\;+\pi \rho_{r}\cos[\left(\omega_{a}-\nu_{3}\right)t+\delta_{W}-\delta_{\phi }-\varepsilon_{3}]+\pi \rho_{r}\cos[\left(\omega_{a}+\nu_{3}\right)t+\delta_{W}+\delta_{\phi }-\varepsilon_{3}]\;+m_{T}R_{T}L\rho_{T}\cos\left[\left(\omega_{a}+\nu_{3}\right)t+\delta_{W_{T}}+\delta_{\phi }-\varepsilon_{3}\right]\;+\left.m_{T}R_{T}L\rho_{T}\cos\left[\left(\omega_{a}+\nu_{3}\right)t+\delta_{W_{T}}+\delta_{\phi }-\varepsilon_{3}\right]\right\}\;+\frac{2P_{0}i\omega_{a}\cos^{2}\beta_{0}I_{\Pi }\left(R\omega_{0}\sin2\beta_{0}+H\cos\beta_{0}\right)}{HBR}\;\times \left\{D\left(\nu_{2}\right)\nu_{2}\rho_{r}\rho_{\psi }\cos\left[\left(\omega_{a}-\nu_{2}\right)t+\delta_{W}-\delta_{\psi }-\varepsilon_{2}\right]\right.\;+D\left(\nu_{2}\right)\nu_{2}\rho_{r}\rho_{\psi }\cos\left[\left(\omega_{a}+\nu_{2}\right)t+\delta_{W}+\delta_{\psi }-\varepsilon_{2}\right]\;+D\left(\nu_{1}\right)\omega_{0}\rho_{r}\rho_{\theta }\sin\left[\left(\omega_{a}-\nu_{1}\right)t+\delta_{W}-\delta_{\theta }-\varepsilon_{1}\right]\;+\left.D\left(\nu_{1}\right)\omega_{0}\rho_{r}\rho_{\theta }\sin\left[\left(\omega_{a}+\nu_{1}\right)t+\delta_{W}+\delta_{\theta }-\varepsilon_{1}\right]\right\}\;+\frac{P_{0}D\left(\nu_{3}\right)i\omega_{a}\cos\beta_{0}}{HBR}\nu_{3}^{2}\rho_{\phi }\left\{I_{\Pi }\rho_{\tau }\sin\left[\left(\omega_{a}-\nu_{3}\right)t+\delta_{V}-\delta_{\phi }-\varepsilon_{3}\right]\right.\;+I_{\Pi }\rho_{\tau }\sin\left[\left(\omega_{a}+\nu_{3}\right)t+\delta_{V}+\delta_{\phi }-\varepsilon_{3}\right]\;+m_{T}R_{T}L\rho_{T}\sin\left[\left(\omega_{a}-\nu_{3}\right)t+\delta_{W_{T}}-\delta_{\phi }-\varepsilon_{3}\right]\;+\left.m_{T}R_{T}L\rho_{T}\sin\left[\left(\omega_{a}+\nu_{3}\right)t+\delta_{W_{T}}+\delta_{\phi }-\varepsilon_{3}\right]\right\},$$

where $D\left(\nu_{i}\right)={\left\{\;{\left[n^{2}-\nu_{i}^{2}\right]}^{2}+4{\left(h-h^{a}\right)}^{2}\nu_{i}^{2}\right\}}^{-\frac{1}{2}}$; $\varepsilon_{i}=arctg\frac{2\left(h-h^{a}\right)}{n^{2}-\nu_{i}^{2}}\nu_{i},$ i=1,2,3.

Thus, in contrast to the case when the gyroscope is affected only by pitch, with the combined influence of acoustic radiation and angular motion of the rocket body in the first approximation, it is possible to establish the conditions of resonant phenomena and assess their nature.

The constant term in Expression (30) is the most interesting. Evidently, the housing pitch will not lead to this, it will only cause the periodic components of the frequency of the angular motion of the rocket (the first three terms in Expression (30)) in the output signal of the device. At the same time, when the frequencies of the penetrating acoustic radiation $\omega_{a}$ and the pitch of the LV body coincide, that is, when $\omega_{a}=\nu$, in the right part of Expression (30), the periodic terms of the total $\left(\omega_{a}+\nu \right)$ frequency and the constant component $\beta_{{}_{1}}^{0}$ appear:

(33) $$\beta_{1}^{0}={\left\{\;{\left[n^{2}-\nu^{2}\right]}^{2}+4{\left(h-h^{a}\right)}^{2}\nu^{2}\right\}}^{-\frac{1}{2}}\;\times \left\{-\frac{P_{0}\omega_{a}^{2}\cos\beta_{0}}{HBR}\nu \rho_{\phi }\left[I_{\Pi }\rho_{\tau }\cos\left(\delta_{V}-\delta_{\phi }-\varepsilon \right)+\pi \rho_{r}\cos\left(\delta_{W}-\delta_{\phi }-\varepsilon \right)\right.\right.\;\left.+m_{T}R_{T}L\rho_{T}\cos\left(\delta_{W_{T}}-\delta_{\phi }-\varepsilon \right)\right]+\frac{2P_{0}i\omega_{a}\cos^{2}\beta_{0}I_{\Pi }\left(R\omega_{0}\sin2\beta_{0}+H\cos\beta_{0}\right)}{HBR}\;\times \left[\nu \rho_{r}\rho_{\psi }\cos\left(\delta_{W}-\delta_{\psi }-\varepsilon \right)+\omega_{0}\rho_{r}\rho_{\theta }\sin\left(\delta_{W}-\delta_{\theta }-\varepsilon \right)\right]+\frac{P_{0}i\omega_{a}\cos\beta_{0}}{HBR}\nu^{2}\rho_{\phi }\;\times \left[I_{\Pi }\rho_{\tau }\sin\left(\delta_{V}-\delta_{\phi }-\varepsilon \right)\right.+\left.\left.m_{T}R_{T}L\rho_{T}\sin\left(\delta_{W_{T}}-\delta_{\phi }-\varepsilon \right)\right]\right\}\;={\left\{\;{\left[n^{2}-\nu^{2}\right]}^{2}+4{\left(h-h^{a}\right)}^{2}\nu^{2}\right\}}^{-\frac{1}{2}}\;\times \frac{P_{0}\omega_{a}\cos\beta_{0}}{HBR}\left\{-\omega_{a}\nu \rho_{\phi }\left[I_{\Pi }\rho_{\tau }\cos\left(\delta_{V}-\delta_{\phi }-\varepsilon \right)+\pi \rho_{r}\cos\left(\delta_{W}-\delta_{\phi }-\varepsilon \right)\right.\right.\;\left.+m_{T}R_{T}L\rho_{T}\cos\left(\delta_{W_{T}}-\delta_{\phi }-\varepsilon \right)\;\right]+2i\cos\beta_{0}I_{\Pi }\left(R\omega_{0}\sin2\beta_{0}+H\cos\beta_{0}\right)\rho_{r}\;\times \left[\nu \rho_{\psi }\cos\left(\delta_{W}-\delta_{\psi }-\varepsilon \right)+\omega_{0}\rho_{\theta }\sin\left(\delta_{W}-\delta_{\theta }-\varepsilon \right)\right]\;+i\nu^{2}\rho_{\phi }\left[I_{\Pi }\rho_{\tau }\sin\left(\delta_{V}-\delta_{\phi }-\varepsilon \right)\right.+\left.\left.m_{T}R_{T}L\rho_{T}\sin\left(\delta_{W_{T}}-\delta_{\phi }-\varepsilon \right)\right]\right\}.$$

Thus, in case of frequencies coincidence of acoustic radiation and angular motion of the rocket body, the output signal of the device will contain:

$$\beta =\beta_{0}+\beta_{1}^{\left(0\right)}$$

which already follows from the first approximation. In other words, the LV pitch will emphasize the frequencies of the sound field $\omega_{a}$. The remaining components will add to the range of periodic terms.

### 2.2. The Second Approximation: Synchronous Fuselage Pitch

We turn to the equation of the second approximation (20). The right part here contains harmonic terms and constants. It is obvious that the harmonic terms in the case of asynchronous rocket body pitch will have frequencies of the $\nu_{ij}=\pm \nu_{i}+\nu_{j}$ form with different combinations of signs and indices i and j. In this case, the oscillation amplitudes will be of the second order of smallness. The constant term in the right-hand side of Equation (20) is the most interesting, since in the steady motion of this constant, C will correspond to some shift of the output signal $\beta_{2}^{\left(0\right)}$ in the instrument readings, defined as the frequency solution of Equation (20):

(34) $$n^{2}\beta_{2}^{\left(0\right)}=C;\;\beta_{2}^{\left(0\right)}=\frac{C}{n^{2}}.$$

Thus, the output signal β will contain $\beta =\beta_{0}+\beta_{1}^{\left(0\right)}+\beta_{2}^{\left(0\right)}$, and instead of the measured angular velocity, $\omega_{0}$ will show $\omega_{0}+\Delta \omega_{1}+\Delta \omega_{2}$, where the last two terms correspond to the “false” angular velocity.

Let us move on to the definition of a constant C. In the case of synchronous LV body pitch we have:

$$\begin{array}{c} \langle \omega_{2x}\rangle =-\langle \omega_{z}\psi \rangle =-\underset{T\to \infty }{lim}\frac{1}{T}\int_{0}^{T}\omega_{z}\psi dt=-\nu \rho_{\phi }\rho_{\psi }\underset{T\to \infty }{lim}\left(\frac{1}{T}\right.\int_{0}^{T}\sin\left(\nu t+\delta_{\psi }\right)\\ \times \cos\left(\nu t+\delta_{\phi }\right)\left.dt\right)=-\frac{1}{2}\nu \rho_{\phi }\rho_{\psi }\sin\left(\delta_{\psi }-\delta_{\phi }\right);\\ \langle \omega_{2z}\rangle =\langle \frac{\omega_{0}}{2}\left(\theta^{2}+\psi^{2}\right)-\dot{\psi }\theta \rangle =\frac{\omega_{0}}{2}\langle \left(\theta^{2}+\psi^{2}\right)\rangle -\langle \dot{\psi }\theta \rangle \\ =\frac{1}{2}\omega_{0}\langle \left[\rho_{\theta }^{2}\sin^{2}\left(\nu t+\delta_{\theta }\right)+\rho_{\psi }^{2}\sin^{2}\left(\nu t+\delta_{\psi }\right)\right]\rangle -\nu \rho_{\theta }\rho_{\psi }\langle \left[\cos\left(\nu t+\delta_{\psi }\right)\sin\left(\nu t+\delta_{\theta }\right)\right]\rangle \\ =\frac{1}{4}\omega_{0}\left(\rho_{\theta }^{2}+\rho_{\psi }^{2}\right)-\frac{1}{2}\nu \rho_{\theta }\rho_{\psi }\sin\left(\delta_{\theta }-\delta_{\psi }\right);\\ \langle \omega_{2y}\rangle =\langle \omega_{1z}\theta \rangle =\langle \nu \rho_{\phi }\cos\left(\nu t+\delta_{\phi }\right)\rho_{\theta }\sin\left(\nu t+\delta_{\theta }\right)\rangle =\frac{1}{2}\nu \rho_{\theta }\rho_{\phi }\sin\left(\delta_{\theta }-\delta_{\phi }\right);\\ \langle \beta_{1}\omega_{1x}\rangle =\langle \beta_{1}\dot{\theta }\rangle -\omega_{0}\langle \beta_{1}\psi \rangle \\ =\frac{1}{2}{\left[{\left(n^{2}-\nu^{2}\right)}^{2}+4{\left(h-h^{a}\right)}^{2}\nu^{2}\right]}^{-\frac{1}{2}}\left\{\left[\left(r-\omega_{0}\right)\nu^{2}\rho_{\theta }^{2}\cos\varepsilon -\left(\omega_{0}r-\nu^{2}\right)\nu \rho_{\theta }\rho_{\psi }\right.\right.\\ \times \sin\left(\delta_{\psi }-\delta_{\theta }-\varepsilon \right)-q\nu^{2}\rho_{\theta }\rho_{\phi }\cos\left(\delta_{\phi }-\delta_{\theta }-\varepsilon \right)]-\frac{1}{2}[\left(r-\omega_{0}\right)\nu \rho_{\theta }\rho_{\psi }\\ \times \sin\left(\delta_{\psi }-\delta_{\theta }+\varepsilon \right)\left.\left.-\left(\omega_{0}r-\nu^{2}\right)\rho_{\psi }^{2}\cos\varepsilon -q\nu \rho_{\psi }\rho_{\phi }\sin\left(\delta_{\psi }-\delta_{\phi }+\varepsilon \right)\right]\right\};\\ \langle \beta_{1}\omega_{1z}\rangle =\frac{1}{2}[\left(r-\omega_{0}\right)\nu^{2}\rho_{\theta }\rho_{\phi }\cos\left(\delta_{\phi }-\delta_{\theta }+\varepsilon \right)\\ -\left(\omega_{0}r-\nu^{2}\right)\nu \rho_{\phi }\rho_{\psi }\sin\left(\delta_{\psi }-\delta_{\phi }-\varepsilon \right)-q\nu^{2}\rho_{\phi }^{2}\cos\varepsilon ]{\left[{\left(n^{2}-\nu^{2}\right)}^{2}+4{\left(h-h^{a}\right)}^{2}\nu^{2}\right]}^{-\frac{1}{2}};\\ \langle \beta_{1}l^{\prime }\omega_{1y}\rangle :\\ l^{\prime }=\frac{l^{a}}{B}=\frac{R\omega_{0}\cos2\beta_{0}\cos\beta_{0}\left(2-tg2\beta_{0}tg\beta_{0}\right)-H\sin2\beta_{0}}{HBR}\\ \times 4P_{0}i\omega_{a}I_{\Pi }\rho_{r}\cos\left(\omega_{a}t+\delta_{W}\right);\\ l^{\prime }\omega_{1y}=\frac{R\omega_{0}\cos2\beta_{0}\cos\beta_{0}\left(2-tg2\beta_{0}tg\beta_{0}\right)-H\sin2\beta_{0}}{HBR}2P_{0}i\omega_{a}I_{\Pi }\rho_{r}\\ \times \left\{\nu \rho_{\psi }\sin\left[\;\left(\omega_{a}-\nu \right)t+\delta_{W}-\delta_{\psi }\right]+\nu \right.\rho_{\psi }\sin\left[\left(\omega_{a}+\nu \right)t+\delta_{W}+\delta_{\psi }\right]\\ \left.-\omega_{0}\rho_{\theta }\sin\left[\left(\omega_{a}-\nu \right)t+\delta_{W}-\delta_{\theta }\right]+\omega_{0}\rho_{\theta }\sin\left[\left(\omega_{a}+\nu \right)t+\delta_{W}+\delta_{\theta }\right]\right\};\\ \langle \beta_{1}l^{\prime }\omega_{1y}\rangle =\\ ={\left[{\left(n^{2}-\nu^{2}\right)}^{2}+4{\left(h-h^{a}\right)}^{2}\nu^{2}\right]}^{-\frac{1}{2}}\frac{R\omega_{0}\cos2\beta_{0}\cos\beta_{0}\left(2-tg2\beta_{0}tg\beta_{0}\right)-H\sin2\beta_{0}}{HBR}\\ \times 2P_{0}i\omega_{a}I_{\Pi }\rho_{r}\{\{-\frac{P_{0}\omega_{a}^{2}\cos\beta_{0}}{2HBR}\nu \rho_{\phi }\{\nu \rho_{\psi }I_{\Pi }\rho_{\tau }\sin\left(\delta_{W}-\delta_{\psi }-\delta_{V}+\delta_{\phi }+\varepsilon \right)\\ +\nu \rho_{\psi }\pi I_{\Pi }\rho_{r}\sin\left(\delta_{W}-\delta_{\psi }-\delta_{W}+\delta_{\phi }+\varepsilon \right)+\nu \rho_{\psi }m_{T}R_{T}L\rho_{T}\sin\left(\delta_{W}-\delta_{\psi }-\delta_{W_{T}}+\delta_{\phi }+\varepsilon \right)\\ -\omega_{0}\rho_{\theta }I_{\Pi }\rho_{\tau }\sin\left(\delta_{W}-\delta_{\theta }-\delta_{V}+\delta_{\phi }+\varepsilon \right)-\omega_{0}\rho_{\theta }\pi I_{\Pi }\rho_{r}\sin\left(\delta_{W}-\delta_{\theta }-\delta_{W}+\delta_{\phi }+\varepsilon \right)\\ -\omega_{0}\rho_{\theta }m_{T}R_{T}L\rho_{T}\sin\left(\delta_{W}-\delta_{\theta }-\delta_{W_{T}}+\delta_{\phi }+\varepsilon \right)\\ +\nu \rho_{\psi }I_{\Pi }\rho_{\tau }\sin\left(\delta_{W}+\delta_{\psi }-\delta_{V}-\delta_{\phi }+\varepsilon \right)+\nu \rho_{\psi }\pi I_{\Pi }\rho_{r}\sin\left(\delta_{W}+\delta_{\psi }-\delta_{W}-\delta_{\phi }+\varepsilon \right)\\ +\nu \rho_{\psi }m_{T}R_{T}L\rho_{T}\sin\left(\delta_{W}+\delta_{\psi }-\delta_{W_{T}}-\delta_{\phi }+\varepsilon \right)\\ +\omega_{0}\rho_{\theta }I_{\Pi }\rho_{\tau }\sin\left(\delta_{W}+\delta_{\theta }-\delta_{V}-\delta_{\phi }+\varepsilon \right)+\omega_{0}\rho_{\theta }\pi I_{\Pi }\rho_{r}\sin\left(\delta_{W}+\delta_{\theta }-\delta_{W}-\delta_{\phi }+\varepsilon \right)\\ \left.+\omega_{0}\rho_{\theta }m_{T}R_{T}L\rho_{T}\sin\left(\delta_{W}+\delta_{\theta }-\delta_{W_{T}}-\delta_{\phi }+\varepsilon \right)\right\}\\ +\frac{P_{0}i\omega_{a}\cos^{2}\beta_{0}I_{\Pi }\left(R\omega_{0}\sin2\beta_{0}+H\cos\beta_{0}\right)}{HBR}\\ \times \left\{\nu^{2}\rho_{\psi }^{2}\rho_{r}\sin\left(\delta_{W}-\delta_{\psi }-\delta_{W}+\delta_{\psi }+\varepsilon \right)+\nu \rho_{\psi }\omega_{0}\rho_{r}\rho_{\theta }\cos\left(\delta_{W}-\delta_{\psi }-\delta_{W}+\delta_{\theta }+\varepsilon \right)\right.\\ -\omega_{0}\rho_{\theta }\nu \rho_{r}\rho_{\psi }\sin\left(\delta_{W}-\delta_{\theta }-\delta_{W}+\delta_{\psi }+\varepsilon \right)-\omega_{0}^{2}\rho_{\theta }^{2}\rho_{r}\cos\left(\delta_{W}-\delta_{\theta }+\delta_{W}+\delta_{\theta }+\varepsilon \right)\\ +\nu^{2}\rho_{\psi }^{2}\rho_{r}\sin\left(\delta_{W}+\delta_{\psi }-\delta_{W}-\delta_{\psi }+\varepsilon \right)+\nu \rho_{\psi }\omega_{0}\rho_{r}\rho_{\theta }\cos\left(\delta_{W}+\delta_{\psi }-\delta_{W}-\delta_{\theta }+\varepsilon \right)\\ \left.+\omega_{0}\rho_{\theta }\nu \rho_{r}\rho_{\psi }\sin\left(\delta_{W}+\delta_{\theta }-\delta_{W}-\delta_{\psi }+\varepsilon \right)+\omega_{0}^{2}\rho_{\theta }^{2}\rho_{r}\cos\left(\delta_{W}+\delta_{\theta }-\delta_{W}-\delta_{\theta }+\varepsilon \right)\right\}\\ +\frac{P_{0}i\omega_{a}\cos\beta_{0}}{2HBR}\nu^{2}\rho_{\phi }\left\{\nu \rho_{\psi }I_{\Pi }\rho_{\tau }\cos\left(\delta_{W}-\delta_{\psi }-\delta_{V}+\delta_{\phi }+\varepsilon \right)\right.+\nu \rho_{\psi }m_{T}R_{T}L\rho_{T}\\ \times \cos\left(\delta_{W}-\delta_{\psi }-\delta_{W_{T}}+\delta_{\phi }+\varepsilon \right)-\omega_{0}\rho_{\theta }I_{\Pi }\rho_{\tau }\cos(\delta_{W}-\delta_{\theta }-\delta_{V}+\delta_{\phi }+\varepsilon )\\ -\omega_{0}\rho_{\theta }m_{T}R_{T}L\rho_{T}\cos\left(\delta_{W}-\delta_{\theta }-\delta_{W_{T}}+\delta_{\phi }+\varepsilon \right)+\nu \rho_{\psi }I_{\Pi }\rho_{\tau }\cos(\delta_{W}+\delta_{\psi }-\delta_{V}\\ -\delta_{\phi }+\varepsilon )+\nu \rho_{\psi }m_{T}R_{T}L\rho_{T}\cos\left(\delta_{W}+\delta_{\psi }-\delta_{W_{T}}-\delta_{\phi }+\varepsilon \right)+\omega_{0}\rho_{\theta }I_{\Pi }\rho_{\tau }\\ \times \cos\left(\delta_{W}+\delta_{\theta }-\delta_{V}-\delta_{\phi }+\varepsilon \right)\left.\left.\left.+\omega_{0}\rho_{\theta }m_{T}R_{T}L\rho_{T}\cos\left(\delta_{W}+\delta_{\theta }-\delta_{W_{T}}-\delta_{\phi }+\varepsilon \right)\right\}\right\}\right\}\\ ={\left[{\left(n^{2}-\nu^{2}\right)}^{2}+4{\left(h-h^{a}\right)}^{2}\nu^{2}\right]}^{-\frac{1}{2}}\\ \times \frac{R\omega_{0}\cos2\beta_{0}\cos\beta_{0}\left(2-tg2\beta_{0}tg\beta_{0}\right)-H\sin2\beta_{0}}{HBR}2P_{0}i\omega_{a}I_{\Pi }\rho_{r}\\ \times \left\{\left\{-\frac{P_{0}\omega_{{}_{a}}^{2}\cos\beta_{0}}{2HBR}\nu \rho_{\phi }\left\{2\nu \rho_{\psi }I_{\Pi }\rho_{\tau }\sin\left(\delta_{W}-\delta_{V}+\varepsilon \right)\cos\left(\delta_{\phi }-\delta_{\psi }\right)\right.\right.\right.\\ +2\omega_{0}\rho_{\theta }I_{\Pi }\rho_{\tau }\sin\left(\delta_{\theta }-\delta_{\phi }\right)\cos\left(\delta_{W}-\delta_{V}+\varepsilon \right)+2\pi I_{\Pi }\nu \rho_{\psi }\rho_{r}\sin\varepsilon \cos\left(\delta_{\psi }-\delta_{\phi }\right)\\ +2\nu m_{T}R_{T}L\rho_{T}\rho_{\psi }\sin\left(\delta_{W}-\delta_{W_{T}}+\varepsilon \right)\cos\left(\delta_{\psi }-\delta_{\phi }\right)+2\pi I_{\Pi }\omega_{0}\rho_{\theta }\rho_{r}\sin\left(\delta_{\theta }-\delta_{\phi }\right)\\ \times \cos\varepsilon +\left.2\omega_{0}\rho_{\theta }m_{T}R_{T}L\rho_{T}\sin\left(\delta_{\theta }-\delta_{\phi }\right)\cos\left(\delta_{W}-\delta_{W_{T}}+\varepsilon \right)\right\}\\ +\frac{P_{0}i\omega_{a}\cos^{2}\beta_{0}I_{\Pi }\left(R\omega_{0}\sin2\beta_{0}+H\cos\beta_{0}\right)}{HBR}\\ \times \left\{2\nu^{2}\rho_{r}\rho_{{}_{\psi }}^{2}\sin\varepsilon +2\nu \omega_{0}\rho_{\theta }\rho_{\psi }\rho_{r}\cos\left(\delta_{\theta }-\delta_{\psi }+\varepsilon \right)\right.+2\nu \omega_{0}\rho_{\theta }\rho_{\psi }\rho_{r}\cos\frac{\pi }{4}\\ \times \sin(\frac{\pi }{4}-\delta_{\psi }+\delta_{\theta }-\varepsilon )\}+\frac{P_{0}i\omega_{a}\cos\beta_{0}}{2HBR}\nu^{2}\rho_{\phi }\{2\nu \rho_{\psi }\rho_{\tau }I_{\Pi }\cos\left(\delta_{W}-\delta_{V}+\varepsilon \right)\\ \times \cos\left(\delta_{\phi }-\delta_{\psi }\right)+2\nu \rho_{\psi }\rho_{T}m_{T}R_{T}L\cos\left(\delta_{W}-\delta_{W_{T}}+\varepsilon \right)\cos\left(\delta_{\phi }-\delta_{\psi }\right)+2\omega_{0}I_{\Pi }\rho_{\theta }\rho_{\tau }\\ \times \sin\left(\delta_{W}-\delta_{V}+\varepsilon \right)\sin\left(\delta_{\theta }-\delta_{\phi }\right)+2\omega_{0}m_{T}R_{T}L\rho_{\theta }\rho_{T}\sin\left(\delta_{W}-\delta_{W_{T}}+\varepsilon \right)\sin\left(\delta_{\theta }-\delta_{\phi }\right)\}\}\}\\ ={\left[{\left(n^{2}-\nu^{2}\right)}^{2}+4{\left(h-h^{a}\right)}^{2}\nu^{2}\right]}^{-\frac{1}{2}}\\ \times \frac{R\omega_{0}\cos2\beta_{0}\cos\beta_{0}\left(2-tg2\beta_{0}tg\beta_{0}\right)-H\sin2\beta_{0}}{HBR}2P_{0}i\omega_{a}I_{\Pi }\rho_{r}\\ \times \left\{\left\{-\frac{P_{0}\omega_{{}_{a}}^{2}\cos\beta_{0}}{HBR}\nu \rho_{\phi }\left\{\nu I_{\Pi }\rho_{\psi }\rho_{\tau }\sin\left(\delta_{W}-\delta_{V}+\varepsilon \right)\cos\left(\delta_{\phi }-\delta_{\psi }\right)\right.\right.\right.\\ +\omega_{0}I_{\Pi }\rho_{\theta }\rho_{\tau }\sin\left(\delta_{W}-\delta_{V}+\varepsilon \right)\cos\left(\delta_{\theta }-\delta_{\phi }\right)+\pi \nu I_{\Pi }\rho_{\psi }\rho_{r}\sin\varepsilon \cos\left(\delta_{\phi }-\delta_{\psi }\right)\\ +\nu m_{T}R_{T}L\rho_{T}\rho_{\psi }\sin\left(\delta_{W}-\delta_{W_{T}}+\varepsilon \right)\cos\left(\delta_{\phi }-\delta_{\psi }\right)+\pi \omega_{0}I_{\Pi }\rho_{\theta }\rho_{r}\cos\varepsilon \sin\left(\delta_{\theta }-\delta_{\phi }\right)\\ +\omega_{0}m_{T}R_{T}L\rho_{\theta }\rho_{T}\cos\left(\delta_{W}-\delta_{W_{T}}+\varepsilon \right)\sin\left(\delta_{\theta }-\delta_{\phi }\right)\}\\ +\frac{2P_{0}i\omega_{a}\cos^{2}\beta_{0}I_{\Pi }\left(R\omega_{0}\sin2\beta_{0}+H\cos\beta_{0}\right)}{HBR}\nu \rho_{r}\rho_{\psi }[\nu \rho_{\psi }\sin\varepsilon +\omega_{0}\rho_{\theta }\\ \times \cos\left(\delta_{\theta }-\delta_{\psi }+\varepsilon \right)+\omega_{0}\rho_{\theta }\sin(\frac{\pi }{4}-\delta_{\psi }+\delta_{\theta }-\varepsilon )\cos\frac{\pi }{4}]\\ +\frac{P_{0}i\omega_{a}\cos\beta_{0}}{HBR}\nu^{2}\rho_{\phi }\left[\nu I_{\Pi }\rho_{\tau }\rho_{\psi }\cos\left(\delta_{W}-\delta_{V}+\varepsilon \right)\cos\left(\delta_{\phi }-\delta_{\psi }\right)\right.\\ +\nu \rho_{\psi }\rho_{T}m_{T}R_{T}L\cos\left(\delta_{W}-\delta_{W_{T}}+\varepsilon \right)\cos\left(\delta_{\phi }-\delta_{\psi }\right)+\omega_{0}I_{\Pi }\rho_{\theta }\rho_{\tau }\sin\left(\delta_{W}-\delta_{V}+\varepsilon \right)\\ \times \sin\left(\delta_{\theta }-\delta_{\phi }\right)+\omega_{0}m_{T}R_{T}L\rho_{\theta }\rho_{T}\sin(\delta_{W}-\delta_{W_{T}}+\varepsilon )\sin(\delta_{\theta }-\delta_{\phi })]\}\};\\ \langle {\dot{\beta }}_{1}\mu^{\prime }\omega_{1z}\rangle ={\left[{\left(n^{2}-\nu^{2}\right)}^{2}+4{\left(h-h^{a}\right)}^{2}\nu^{2}\right]}^{-\frac{1}{2}}\\ \times \left\{\left\{\frac{P_{0}\omega_{{}_{a}}^{2}\cos\beta_{0}}{2HBR}\nu \rho_{\phi }\right.\right.\left\{-2\omega_{a}(I_{{}_{\Pi }}^{2}\rho_{{}_{\tau }}^{2}+\pi^{2}I_{\Pi }^{2}\rho_{r}^{2}+m_{T}^{2}R_{T}^{2}L^{2})\sin\varepsilon \right.\\ +2\omega_{a}\pi I_{\Pi }^{2}\rho_{r}\rho_{\tau }\sin\left(\delta_{W}-\delta_{V}-\varepsilon \right)+2\omega_{a}m_{T}R_{T}L\rho_{T}I_{\Pi }\rho_{\tau }\sin\left(\delta_{W_{T}}-\delta_{V}-\varepsilon \right)\\ +2\omega_{a}m_{T}R_{T}L\rho_{T}\pi I_{\Pi }\rho_{r}\sin\left(\delta_{W}-\delta_{W_{T}}-\varepsilon \right)\\ +I_{\Pi }\rho_{\tau }\left(\omega_{a}-\nu \right)\left(\pi I_{\Pi }\rho_{r}+m_{T}R_{T}L\rho_{T}\right)\sin\left(\delta_{V}-\varepsilon \right)\\ +\pi I_{{}_{\Pi }}^{2}\rho_{r}\rho_{\tau }(\omega_{a}+\nu )\sin(\delta_{V}-\delta_{W}-\varepsilon )+I_{\Pi }\rho_{\tau }m_{T}R_{T}L\rho_{T}(\omega_{a}+\nu )\sin(\delta_{V}-\delta_{W_{T}}-\varepsilon )\}\\ -\frac{P_{0}i\omega_{a}\cos^{2}\beta_{0}I_{\Pi }\left(R\omega_{0}\sin2\beta_{0}+H\cos\beta_{0}\right)}{HBR}\\ \times \{I_{\Pi }\rho_{r}\rho_{\tau }\rho_{\psi }\nu (\omega_{a}-\nu )\sin(\delta_{W}-\delta_{V}-\delta_{\psi }+\delta_{\phi }-\varepsilon )\\ -I_{\Pi }\rho_{r}\rho_{\tau }\rho_{\theta }\omega_{0}(\omega_{a}-\nu )\cos(\delta_{W}-\delta_{V}-\delta_{\theta }+\delta_{\phi }-\varepsilon )\\ +I_{\Pi }\rho_{r}\rho_{\tau }\rho_{\psi }\nu \left(\omega_{a}+\nu \right)\sin\left(\delta_{W}+\delta_{\psi }-\delta_{V}-\delta_{\phi }-\varepsilon \right)\\ -I_{\Pi }\rho_{r}\rho_{\tau }\rho_{\psi }\omega_{0}\left(\omega_{a}+\nu \right)\cos\left(\delta_{W}-\delta_{V}+\delta_{\theta }-\delta_{\phi }-\varepsilon \right)\\ +I_{\Pi }\rho_{{}_{r}}^{2}\rho_{\psi }\nu \left(\omega_{a}-\nu \right)\sin\left(\delta_{\phi }-\delta_{\psi }-\varepsilon \right)-\pi I_{\Pi }\rho_{{}_{r}}^{2}\rho_{\theta }\omega_{0}\left(\omega_{a}-\nu \right)\cos\left(\delta_{\phi }-\delta_{\theta }-\varepsilon \right)\\ +\pi I_{\Pi }\rho_{{}_{r}}^{2}\rho_{\psi }\nu \left(\omega_{a}+\nu \right)\sin\left(\delta_{\psi }-\delta_{\phi }-\varepsilon \right)-\pi I_{\Pi }\rho_{{}_{r}}^{2}\rho_{\theta }\omega_{0}\left(\omega_{a}+\nu \right)\cos\left(\delta_{\theta }-\delta_{\phi }-\varepsilon \right)\\ +m_{T}R_{T}L\rho_{r}\rho_{\tau }\rho_{\psi }\nu \left(\omega_{a}-\nu \right)\sin\left(\delta_{W}-\delta_{W_{T}}-\delta_{\psi }-\varepsilon \right)\\ -m_{T}R_{T}L\rho_{r}\rho_{\theta }\rho_{T}\omega_{0}\left(\omega_{a}-\nu \right)\cos\left(\delta_{W}-\delta_{W_{T}}-\delta_{\theta }-\delta_{\phi }-\varepsilon \right)\\ +m_{T}R_{T}L\rho_{r}\rho_{\psi }\rho_{T}\nu \left(\omega_{a}+\nu \right)\sin\left(\delta_{W}-\delta_{W_{T}}+\delta_{\psi }-\delta_{\phi }-\varepsilon \right)\\ -m_{T}R_{T}L\rho_{r}\rho_{\theta }\rho_{T}\omega_{0}\left(\omega_{a}+\nu \right)\cos\left(\delta_{W}-\delta_{W_{T}}+\delta_{\theta }-\delta_{\phi }-\varepsilon \right)\}\\ +\frac{2P_{0}i\omega_{{}_{a}}^{2}\cos\beta_{0}}{HBR}\nu^{2}\rho_{\phi }\{(I_{{}_{\Pi }}^{2}\rho_{{}_{\tau }}^{2}+m_{T}^{2}R_{T}^{2}L^{2}\rho_{T}^{2})\cos\varepsilon +\pi I_{\Pi }^{2}\rho_{r}\rho_{\tau }\cos(\delta_{V}-\delta_{W}-\varepsilon )\\ +m_{T}R_{T}LI_{\Pi }\rho_{\tau }\rho_{T}\cos\left(\delta_{V}-\delta_{W_{T}}-\varepsilon \right)+m_{T}R_{T}LI_{\Pi }\rho_{\tau }\rho_{T}\cos\left(\delta_{W_{T}}-\delta_{V}-\varepsilon \right)\\ +\pi m_{T}R_{T}LI_{\Pi }\rho_{r}\rho_{T}\cos(\delta_{W_{T}}-\delta_{W}-\varepsilon )\}\}\};\\ \langle {\dot{\beta }}_{1}\mu \omega_{0}\beta_{1}\rangle =\omega_{0}\langle {\dot{\beta }}_{1}\beta_{1}\mu \rangle =0;\\ \langle \frac{1}{2}\omega_{0}q^{a}\beta_{{}_{1}}^{2}\rangle =\frac{1}{2}\omega_{0}\langle q^{a}\beta_{{}_{1}}^{2}\rangle =0;\\ q^{a}=\frac{q_{1}^{a}}{B}=\frac{-2P_{0}\omega_{{}_{a}}^{2}\cos\beta_{0}}{HBR}\left\{I_{\Pi }\left[\rho_{\tau }\cos\left(\omega_{a}t+\delta_{V}\right)+\rho_{r}\cos\left(\omega_{a}t+\delta_{W}\right)\right]\right.\\ +\left.m_{T}R_{T}L\rho_{T}\cos\left(\omega_{a}t+\delta_{W_{T}}\right)\right\};\\ \frac{1}{2}\omega_{0}\langle q^{\prime \prime }\beta_{{}_{1}}^{2}\rangle ;\\ q^{\prime \prime }=\frac{{q^{\prime \prime }}_{1}}{B}=\frac{\left(-2R\omega_{0}\sin2\beta_{0}+H\cos\beta_{0}\right)}{B};\\ \langle \beta_{{}_{1}}^{2}\rangle =\langle [{\left(n^{2}-\nu^{2}\right)}^{2}+4{\left(h-h^{a}\right)}^{2}\nu^{2}]\\ \times \{\{{\left(r-\omega_{0}\right)}^{2}\nu^{2}\rho_{\theta }^{2}\cos^{2}(\nu t+\delta_{\theta }-\varepsilon )-{\left(r\omega_{0}-\nu^{2}\right)}^{2}\rho_{\psi }^{2}\sin(\nu t+\delta_{\psi }-\varepsilon )\\ -q^{2}\nu^{2}\rho_{\phi }^{2}\cos^{2}\left(\nu t+\delta_{\phi }-\varepsilon \right)-\left(r\omega_{0}-\nu^{2}\right)\nu q\rho_{\psi }\rho_{\phi }[\sin\left(\delta_{\psi }-\delta_{\phi }\right)\\ +\cos\left(2\nu t+\delta_{\psi }+\delta_{\phi }-2\varepsilon \right)]-\left(r-\omega_{0}\right)\left(r\omega_{0}-\nu^{2}\right)\nu \rho_{\theta }\rho_{\psi }[\sin\left(\delta_{\psi }-\delta_{\theta }\right)\\ +\cos\left(2\nu t+\delta_{\psi }+\delta_{\theta }-2\varepsilon \right)]-\left(r-\omega_{0}\right)\nu^{2}q\rho_{\theta }\rho_{\phi }[\cos\left(\delta_{\phi }-\delta_{\theta }\right)\\ +\cos\left(2\nu t+\delta_{\theta }+\delta_{\phi }-2\varepsilon \right)]\\ -\frac{P_{0}\omega_{{}_{a}}^{2}\cos\beta_{0}}{HBR}\nu \rho_{\phi }\frac{2P_{0}i\omega_{a}\cos^{2}\beta_{0}I_{\Pi }\left(R\omega_{0}\sin2\beta_{0}+H\cos\beta_{0}\right)}{HBR}\rho_{r}\\ \times \left\{I_{\Pi }\rho_{\tau }\right.[\nu \rho_{\psi }\cos\left(\delta_{V}-\delta_{\phi }-\delta_{W}+\delta_{\psi }\right)-\omega_{0}\rho_{\theta }\sin\left(\delta_{W}-\delta_{\theta }-\delta_{V}+\delta_{\phi }\right)\\ +\nu \rho_{\psi }\cos\left(\delta_{V}+\delta_{\phi }-\delta_{W}-\delta_{\psi }\right)+\omega_{0}\rho_{\theta }\sin\left(\delta_{W}-\delta_{\theta }-\delta_{V}-\delta_{\phi }\right)]\\ +\pi I_{\Pi }[\nu \rho_{\psi }\cos\left(\delta_{\psi }-\delta_{\phi }\right)+\omega_{0}\rho_{\theta }\sin\left(\delta_{\phi }-\delta_{\theta }\right)+\nu \rho_{\psi }\cos\left(\delta_{\phi }-\delta_{\psi }\right)+\omega_{0}\rho_{\theta }\\ \times \sin\left(\delta_{\theta }-\delta_{\phi }\right)]+m_{T}R_{T}L\rho_{T}[\nu \rho_{\psi }\cos\left(\delta_{W_{T}}-\delta_{\phi }-\delta_{W}+\delta_{\psi }\right)+\omega_{0}\rho_{\theta }\sin(\delta_{W}-\delta_{\theta }\\ -\delta_{W_{T}}+\delta_{\phi })+\nu \rho_{\psi }\cos\left(\delta_{W_{T}}+\delta_{\phi }-\delta_{W}-\delta_{\psi }\right)+\omega_{0}\rho_{\theta }\sin\left(\delta_{W}+\delta_{\theta }-\delta_{W_{T}}-\delta_{\phi }\right)]\}\\ -\frac{P_{0}\omega_{{}_{a}}^{2}\cos\beta_{0}}{HBR}\nu \rho_{\phi }\frac{P_{0}i\omega_{a}\cos\beta_{0}}{HBR}\nu^{2}\rho_{\phi }\\ \times \left\{I_{\Pi }\rho_{\tau }\right.\rho_{T}m_{T}R_{T}L\sin\left(\delta_{W_{T}}-\delta_{V}\right)+\pi I_{\Pi }\rho_{r}\left[I_{\Pi }\rho_{\tau }\sin\left(\delta_{V}-\delta_{W}\right)\right.\\ +m_{T}R_{T}L\rho_{T}\sin\left(\delta_{W_{T}}-\delta_{W}\right)]+m_{T}R_{T}L\rho_{T}I_{\Pi }\rho_{\tau }\sin\left(\delta_{V}-\delta_{W_{T}}\right)\\ +I_{\Pi }\rho_{\tau }\rho_{T}m_{T}R_{T}L\sin\left(\delta_{W_{T}}-\delta_{V}\right)+m_{T}R_{T}L\rho_{T}I_{\Pi }\rho_{\tau }\sin\left(\delta_{V}-\delta_{W_{T}}\right)\\ +\left.\pi I_{\Pi }\rho_{r}[I_{\Pi }\rho_{\tau }\sin\left(\delta_{V}-\delta_{W}\right)+m_{T}R_{T}L\rho_{T}\sin\left(\delta_{W_{T}}-\delta_{W}\right)]\right\}\\ +\frac{P_{0}i\omega_{a}\cos^{2}\beta_{0}I_{\Pi }\left(R\omega_{0}\sin2\beta_{0}+H\cos\beta_{0}\right)}{HBR}\frac{P_{0}i\omega_{a}\cos\beta_{0}}{HBR}\nu^{2}\rho_{\phi }\rho_{r}\\ \times \left\{\nu \rho_{\psi }I_{\Pi }\rho_{\tau }[\sin\left(\delta_{V}-\delta_{\phi }-\delta_{W}+\delta_{\psi }\right)+\sin\left(\delta_{V}+\delta_{\phi }-\delta_{W}-\delta_{\psi }\right)]\right.\\ +\omega_{0}\rho_{\theta }I_{\Pi }\rho_{\tau }[\cos\left(\delta_{W}-\delta_{\theta }-\delta_{V}+\delta_{\phi }\right)+\cos\left(\delta_{W}+\delta_{\theta }-\delta_{V}-\delta_{\phi }\right)]\\ +m_{T}R_{T}L\rho_{T}\nu \rho_{\psi }[\sin\left(\delta_{W_{T}}-\delta_{\phi }-\delta_{W}+\delta_{\psi }\right)+\sin\left(\delta_{W_{T}}+\delta_{\phi }-\delta_{W}-\delta_{\psi }\right)]\\ +m_{T}R_{T}L\rho_{T}\omega_{0}\rho_{\theta }[\cos\left(\delta_{W}-\delta_{\theta }-\delta_{W_{T}}+\delta_{\phi }\right)+\cos\left(\delta_{W}+\delta_{\theta }-\delta_{W_{T}}-\delta_{\phi }\right)]\\ +\frac{P_{0}{}^{2}\omega_{{}_{a}}^{4}\cos^{2}\beta_{0}}{H^{2}B^{2}R^{2}}\nu^{2}\rho_{{}_{\phi }}^{2}\\ \times \left\{\left\{I_{{}_{\Pi }}^{2}\rho_{\tau }^{2}\left\{\cos^{2}\right.[\left(\omega_{a}-\nu \right)t+\delta_{V}-\delta_{\phi }-\varepsilon ]\right.+\cos^{2}[\left(\omega_{a}+\nu \right)t+\delta_{V}+\delta_{\phi }-\varepsilon ]\right.\\ +2\cos[\left(\omega_{a}-\nu \right)t+\delta_{V}-\delta_{\phi }-\varepsilon ]\cos[\left(\omega_{a}+\nu \right)\;t+\delta_{V}+\delta_{\phi }-\varepsilon ]\}\\ +\pi^{2}I_{{}_{\Pi }}^{2}\rho_{{}_{r}}^{2}\left\{\cos^{2}\right.[\left(\omega_{a}-\nu \right)t+\delta_{W}-\delta_{\phi }-\varepsilon ]+\cos^{2}[\left(\omega_{a}+\nu \right)\;t+\delta_{W}+\delta_{\phi }-\varepsilon ]\\ +2\cos[\left(\omega_{a}-\nu \right)t+\delta_{W}-\delta_{\phi }-\varepsilon ]\cos[\left(\omega_{a}+\nu \right)\;t+\delta_{W}+\delta_{\phi }-\varepsilon ]\}\\ +m_{{}_{T}}^{2}R_{{}_{T}}^{2}L^{2}\rho_{{}_{T}}^{2}{\left\{\cos\right.}^{2}\left[\left(\omega_{a}-\nu \right)t+\delta_{W_{T}}-\delta_{\phi }-\varepsilon \right]+\cos^{2}\left[\left(\omega_{a}+\nu \right)t+\delta_{W_{T}}+\delta_{\phi }-\varepsilon \right]\\ +2\cos[\left(\omega_{a}-\nu \right)t+\delta_{W_{T}}-\delta_{\phi }-\varepsilon ]\cos[\left(\omega_{a}+\nu \right)t+\delta_{W_{T}}+\delta_{\phi }-\varepsilon ]\}\\ +2\pi I_{{}_{\Pi }}^{2}\rho_{\tau }\rho_{r}\{\cos[(\omega_{a}-\nu )t+\delta_{V}-\delta_{\phi }-\varepsilon ]\cos[(\omega_{a}-\nu )t+\delta_{W}-\delta_{\phi }-\varepsilon ]\\ +\cos[\left(\omega_{a}-\nu \right)t+\delta_{V}-\delta_{\phi }-\varepsilon ]\cos[\left(\omega_{a}+\nu \right)t+\delta_{W}+\delta_{\phi }-\varepsilon ]\\ +\cos[\left(\omega_{a}+\nu \right)t+\delta_{V}+\delta_{\phi }-\varepsilon ]\cos[\left(\omega_{a}-\nu \right)t+\delta_{W}-\delta_{\phi }-\varepsilon ]\\ +\left.\cos[\left(\omega_{a}+\nu \right)t+\delta_{V}+\delta_{\phi }-\varepsilon ]\cos[\left(\omega_{a}+\nu \right)t+\delta_{W}+\delta_{\phi }-\varepsilon ]\right\}\\ +2I_{\Pi }\rho_{\tau }m_{T}R_{T}L\rho_{T}\left\{\cos[\left(\omega_{a}-\nu \right)t+\delta_{V}-\delta_{\phi }-\varepsilon ]\cos[\left(\omega_{a}-\nu \right)t+\delta_{W_{T}}-\delta_{\phi }-\varepsilon ]\right.\\ +\cos[\left(\omega_{a}-\nu \right)t+\delta_{V}-\delta_{\phi }-\varepsilon ]\cos[\left(\omega_{a}+\nu \right)t+\delta_{W_{T}}+\delta_{\phi }-\varepsilon ]\\ +\cos[\left(\omega_{a}+\nu \right)t+\delta_{V}+\delta_{\phi }-\varepsilon ]\cos[\left(\omega_{a}-\nu \right)t+\delta_{W_{T}}-\delta_{\phi }-\varepsilon ]\\ +\cos[\left(\omega_{a}+\nu \right)t+\delta_{V}+\delta_{\phi }-\varepsilon ]\cos[\left(\omega_{a}+\nu \right)t+\delta_{W_{T}}+\delta_{\phi }-\varepsilon ]\}\\ +2\pi I_{\Pi }\rho_{r}m_{T}R_{T}L\rho_{T}\left\{\cos[\left(\omega_{a}-\nu \right)t+\delta_{W}-\delta_{\phi }-\varepsilon ]\cos[\right.\left(\omega_{a}-\nu \right)t+\delta_{W_{T}}-\delta_{\phi }-\varepsilon ]\\ +\cos[\left(\omega_{a}-\nu \right)t+\delta_{W}-\delta_{\phi }-\varepsilon ]\cos[\left(\omega_{a}+\nu \right)t+\delta_{W_{T}}+\delta_{\phi }-\varepsilon ]\\ +\cos[\left(\omega_{a}+\nu \right)t+\delta_{W}+\delta_{\phi }-\varepsilon ]\cos[\left(\omega_{a}-\nu \right)t+\delta_{W_{T}}-\delta_{\phi }-\varepsilon ]\\ +\left.\left.\left.\cos[\left(\omega_{a}+\nu \right)t+\delta_{W}+\delta_{\phi }-\varepsilon ]\cos[\left(\omega_{a}+\nu \right)t+\delta_{W_{T}}+\delta_{\phi }-\varepsilon ]\right\}\right\}\right\}\\ -\frac{4P_{{}_{0}}^{2}\omega_{{}_{a}}^{2}\cos^{4}\beta_{0}I_{{}_{\Pi }}^{2}{\left(R\omega_{0}\sin2\beta_{0}+H\cos\beta_{0}\right)}^{2}\rho_{{}_{r}}^{2}}{H^{2}B^{2}R^{2}}\\ \times \left\{\left\{\nu^{2}\rho_{\psi }^{2}\left\{\cos^{2}\left[\left(\omega_{a}-\nu \right)t+\delta_{W}-\delta_{\psi }-\varepsilon \right]\right.\right.+\cos^{2}\left[\left(\omega_{a}+\nu \right)t+\delta_{W}+\delta_{\psi }-\varepsilon \right]\right.\\ +2\cos[\left(\omega_{a}-\nu \right)t+\delta_{W}-\delta_{\psi }-\varepsilon ]\cos[\left(\omega_{a}+\nu \right)t+\delta_{W}+\delta_{\psi }-\varepsilon ]\}\\ +\omega_{{}_{0}}^{2}\rho_{\theta }^{2}\left\{\cos^{2}\right.\left[\left(\omega_{a}-\nu \right)t+\delta_{W}-\delta_{\theta }-\varepsilon \right]+\cos^{2}\left[\left(\omega_{a}+\nu \right)t+\delta_{W}+\delta_{\theta }-\left.\varepsilon \right]\right.\\ +2\cos\left[\left(\omega_{a}-\nu \right)t+\delta_{W}-\delta_{\theta }-\varepsilon \right]\cos\left.\left[\left(\omega_{a}+\nu \right)t+\delta_{W}+\delta_{\theta }-\varepsilon \right]\right\}\\ +2\nu \rho_{\psi }\omega_{0}\rho_{\theta }\left\{\cos\right.[\left(\omega_{a}-\nu \right)t+\delta_{W}-\delta_{\psi }-\varepsilon ]\cos[\left(\omega_{a}-\nu \right)t+\delta_{W}-\delta_{\theta }-\varepsilon ]\\ +\cos[\left(\omega_{a}-\nu \right)t+\delta_{W}-\delta_{\psi }-\varepsilon ]\cos[\left(\omega_{a}+\nu \right)t+\delta_{W}+\delta_{\theta }-\varepsilon ]\\ +\cos[\left(\omega_{a}+\nu \right)t+\delta_{W}+\delta_{\psi }-\varepsilon ]\cos[\left(\omega_{a}-\nu \right)t+\delta_{W}-\delta_{\theta }-\varepsilon ]\\ +\cos[\left(\omega_{a}+\nu \right)t+\delta_{W}+\delta_{\psi }-\varepsilon ]\cos[\left(\omega_{a}+\nu \right)t+\delta_{W}+\delta_{\theta }-\varepsilon ]\}\}\}\\ -\frac{P_{0}{}^{2}\omega_{{}_{a}}^{2}\cos^{2}\beta_{0}}{H^{2}B^{2}R^{2}}\nu^{4}\rho_{{}_{\phi }}^{2}\\ \times \left\{\left\{I_{{}_{\Pi }}^{2}\rho_{\tau }^{2}\left\{\sin^{2}\right.[\left(\omega_{a}-\nu \right)t+\delta_{V}-\delta_{\phi }-\varepsilon ]\right.+\sin^{2}[\left(\omega_{a}+\nu \right)t+\delta_{V}+\delta_{\phi }-\varepsilon ]\right.\\ +2\sin[\left(\omega_{a}-\nu \right)t+\delta_{V}-\delta_{\phi }-\varepsilon ]\left.\sin[\left(\omega_{a}+\nu \right)t+\delta_{V}+\delta_{\phi }-\varepsilon ]\right\}+m_{T}^{2}R_{T}^{2}L^{2}\rho_{T}^{2}\\ \times \left\{\sin^{2}[\left(\omega_{a}-\nu \right)t+\delta_{W_{T}}-\delta_{\phi }-\varepsilon ]+\sin^{2}\right.[\left(\omega_{a}+\nu \right)t+\delta_{W_{T}}+\delta_{\phi }-\varepsilon ]+2\sin\left[\left(\omega_{a}-\nu \right)t\right.\\ \left.\left.+\delta_{W_{T}}-\delta_{\phi }-\left.\left.\varepsilon \right]\sin[\left(\omega_{a}+\nu \right)t+\delta_{W_{T}}+\delta_{\phi }-\varepsilon ]\right\}\right\}\right\}\rangle =[{\left(n^{2}-\nu^{2}\right)}^{2}+4{\left(h-h^{a}\right)}^{2}\nu^{2}]\\ \times \left\{\left\{\right.\right.\frac{1}{2}{\left(r-\omega_{0}\right)}^{2}\nu^{2}\rho_{\theta }^{2}-\frac{1}{2}q^{2}\nu^{2}\rho_{\phi }^{2}-\left(r\omega_{0}-\nu^{2}\right)\nu q\rho_{\psi }\rho_{\phi }\sin\left(\delta_{\psi }-\delta_{\phi }\right)\\ -\left(r-\omega_{0}\right)\left(r\omega_{0}-\nu^{2}\right)\nu \rho_{\theta }\rho_{\psi }\sin\left(\delta_{\psi }-\delta_{\theta }\right)-\left(r-\omega_{0}\right)\nu^{2}q\rho_{\theta }\rho_{\phi }\cos\left(\delta_{\phi }-\delta_{\theta }\right)\\ -\frac{4P_{0}{}^{2}\omega_{{}_{a}}^{2}\cos^{3}\beta_{0}}{H^{2}B^{2}R^{2}}\nu \rho_{\phi }\rho_{r}i\omega_{a}I_{\Pi }\left(R\omega_{0}\sin2\beta_{0}+H\cos\beta_{0}\right)\\ \times \left\{I_{\Pi }\right.\rho_{\tau }\left[\nu \right.\rho_{\psi }\cos\left(\delta_{V}-\delta_{W}\right)\sin\left(\delta_{\psi }-\delta_{\phi }\right)-\omega_{0}\rho_{\theta }\cos\left(\delta_{W}-\delta_{V}-\delta_{\theta }\right)\left.\sin\delta_{\phi }\right]\\ +\pi I_{\Pi }\left[\nu \rho_{\psi }\cos\left.\left(\delta_{\psi }-\delta_{\phi }\right)\right]+\right.m_{T}R_{T}L\rho_{T}\left[\nu \rho_{\psi }\cos\left(\delta_{W_{T}}-\delta_{W}\right)\right.\cos\left(\delta_{\psi }-\delta_{\phi }\right)\\ +\omega_{0}\rho_{\theta }\sin\left(\delta_{W}-\delta_{W_{T}}\right)\cos\left.\left.\left(\delta_{\phi }-\delta_{\theta }\right)\right]\right\}\\ -2\frac{P_{{}_{0}}^{2}i\omega_{{}_{a}}^{3}\cos^{2}\beta_{0}}{H^{2}B^{2}R^{2}}\nu^{3}\rho_{\phi }^{2}\pi I_{\Pi }\rho_{r}\left[I_{\Pi }\right.\rho_{\tau }\sin\left(\delta_{V}-\delta_{W}\right)+m_{T}R_{T}L\rho_{T}\sin\left.\left(\delta_{W_{T}}-\delta_{W}\right)\right]\\ -\frac{P_{{}_{0}}^{2}\omega_{{}_{a}}^{2}\cos^{3}\beta_{0}I_{\Pi }\left(R\omega_{0}\sin2\beta_{0}+H\cos\beta_{0}\right)}{H^{2}B^{2}R^{2}}\nu^{2}\rho_{\phi }\rho_{r}\left\{2\right.\nu I_{\Pi }\rho_{\psi }\rho_{\tau }\sin\left(\delta_{V}-\delta_{W}\right)\\ \times \cos\left(\delta_{\psi }-\delta_{\phi }\right)+2\omega_{0}I_{\Pi }\rho_{\theta }\rho_{\tau }\cos\left(\delta_{W}-\delta_{V}\right)\cos\left(\delta_{\phi }-\delta_{\theta }\right)+2m_{T}R_{T}L\rho_{T}\nu \rho_{\psi }\\ \times \sin\left(\delta_{W_{T}}-\delta_{W}\right)\cos\left(\delta_{\psi }-\delta_{\phi }\right)+2\omega_{0}m_{T}R_{T}L\rho_{T}\rho_{\theta }\cos\left(\delta_{W}-\delta_{W_{T}}\right)\cos\left.\left(\delta_{\phi }-\delta_{\theta }\right)\right\}\\ +\frac{P_{{}_{0}}^{2}\omega_{{}_{a}}^{4}\cos^{2}\beta_{0}}{H^{2}B^{2}R^{2}}\nu^{2}\rho_{\phi }\left[I_{{}_{\Pi }}^{2}\right.\rho_{\tau }^{2}+\pi^{2}I_{{}_{\Pi }}^{2}\rho_{r}^{2}+m_{{}_{T}}^{2}R_{{}_{T}}^{2}L^{2}\rho_{{}_{T}}^{2}+\pi I_{{}_{\Pi }}^{2}\rho_{\tau }\rho_{r}\\ \times \cos\left(\delta_{V}-\delta_{W}\right)+I_{\Pi }\rho_{\tau }\rho_{T}m_{T}R_{T}L\cos\left(\delta_{V}-\delta_{W_{T}}\right)+\pi I_{\Pi }\rho_{r}\rho_{T}m_{T}R_{T}L\cos\left.\left(\delta_{W}-\delta_{W_{T}}\right)\right]\\ -\frac{4P_{{}_{0}}^{2}\omega_{{}_{a}}^{2}\cos^{4}\beta_{0}I_{{}_{\Pi }}^{2}{\left(R\omega_{0}\sin2\beta_{0}+H\cos\beta_{0}\right)}^{2}}{H^{2}B^{2}R^{2}}\rho_{{}_{r}}^{2}\left[\nu^{2}\rho_{{}_{\psi }}^{2}\right.+\omega_{0}^{2}\rho_{{}_{\theta }}^{2}+2\nu \omega_{0}\rho_{\psi }\rho_{\theta }\\ \times \cos\left.\left(\delta_{\theta }-\delta_{\psi }\right)\right]-\frac{P_{0}{}^{2}\omega_{{}_{a}}^{2}\cos^{2}\beta_{0}}{2H^{2}B^{2}R^{2}}\nu^{4}\rho_{{}_{\phi }}^{2}\left[I_{{}_{\Pi }}^{2}\rho_{\tau }^{2}\right.+m_{{}_{T}}^{2}R_{{}_{T}}^{2}L^{2}\left.\left.\left.\rho_{{}_{T}}^{2}\right]\right\}\right\};\\ \frac{1}{2}\omega_{0}\langle q^{\prime \prime }\beta_{{}_{1}}^{2}\rangle =\frac{\omega_{0}\left(-2R\omega_{0}\sin2\beta_{0}+H\cos\beta_{0}\right)}{2B}\langle \beta_{{}_{1}}^{2}\rangle ;\\ \frac{1}{2}\omega_{0}\langle q^{a}\beta_{{}_{1}}^{2}\rangle =0;q^{a}=\frac{q_{1}^{a}}{B}=\frac{i\omega_{a}\sin\beta_{0}}{B}Q\\ =\frac{i\omega_{a}\sin\beta_{0}}{B}\frac{2P_{0}i\omega_{a}}{HR}\left[I_{\Pi }\left(\rho_{\tau }\cos\left(\omega_{a}t+\left.\delta_{V}\right)\right.\right.\right.+\pi \rho_{r}\cos\left.\left(\omega_{a}t+\delta_{W}\right)\right)\\ +m_{T}R_{T}L\rho_{T}\cos\left(\omega_{a}t+\delta_{W_{T}}\right)\left.\right];\\ \langle \omega_{1x}^{2}\rangle =\langle {\dot{\theta }}^{2}\rangle +\omega_{0}^{2}\langle \psi^{2}\rangle -2\omega_{0}\langle \dot{\theta }\psi \rangle =\frac{1}{2}\nu^{2}\rho_{\theta }^{2}+\frac{1}{2}\omega_{0}^{2}\rho_{\psi }^{2}+\omega_{0}\rho_{\theta }\rho_{\psi }\nu \sin\left(\delta_{\theta }-\delta_{\psi }\right);\\ \langle \omega_{1z}^{2}\rangle =\frac{1}{2}\nu^{2}\rho_{\psi }^{2};\\ \langle \lambda^{2}\omega_{1y}^{2}\rangle ;\lambda =Q_{1}\cos\beta_{0}=\frac{4P_{0}i\omega_{a}\cos\beta_{0}I_{\Pi }\rho_{r}}{HR}\cos\left(\omega_{a}t+\left.\delta_{W}\right)\right.;\\ \lambda^{2}=\frac{-16P_{{}_{0}}^{2}\omega_{{}_{a}}^{2}\cos^{2}\beta_{0}}{H^{2}R^{2}}I_{{}_{\Pi }}^{2}\rho_{{}_{r}}^{2}\cos^{2}\left(\omega_{a}t+\left.\delta_{W}\right)\right.;\\ \omega_{1y}=\dot{\psi }+\omega_{0}\theta =\nu \rho_{\psi }\cos\left(\nu t+\delta_{\psi }\right)+\omega_{0}\rho_{\theta }\sin\left(\nu t+\delta_{\theta }\right);\\ \omega_{{}_{1y}}^{2}=\nu^{2}\rho_{\psi }^{2}\cos^{2}\left(\nu t+\delta_{\psi }\right)+\omega_{{}_{0}}^{2}\rho_{{}_{\theta }}^{2}\sin^{2}\left(\nu t+\delta_{\theta }\right)+\nu \omega_{0}\rho_{\theta }\rho_{\psi }\left[\sin\right.\left(\delta_{\theta }-\delta_{\psi }\right)\\ +\cos\left.\left(2\nu t+\delta_{\theta }+\delta_{\psi }\right)\right];\\ \langle \lambda^{2}\omega_{1y}^{2}\rangle =\frac{-16P_{{}_{0}}^{2}\omega_{{}_{a}}^{2}\cos^{2}\beta_{0}}{H^{2}R^{2}}I_{{}_{\Pi }}^{2}\rho_{{}_{r}}^{2}{\langle \nu }^{2}\rho_{\psi }^{2}\cos^{2}\left(\omega_{a}t+\left.\delta_{W}\right)\right.\cos^{2}\left(\nu t+\delta_{\psi }\right)\\ +\omega_{{}_{0}}^{2}\rho_{{}_{\theta }}^{2}\cos^{2}\left(\omega_{a}t+\delta_{W}\right)\sin^{2}\left(\nu t+\delta_{\theta }\right)+\nu \omega_{0}\rho_{\theta }\rho_{\psi }\sin\left(\delta_{\theta }-\delta_{\psi }\right)\\ \times \cos^{2}\left(\omega_{a}t+\delta_{W}\right)+\nu \omega_{0}\rho_{\theta }\rho_{\psi }\cos^{2}\left(\omega_{a}t+\delta_{W}\right)\cos\left(2\nu t+\delta_{\theta }+\delta_{\psi }\right)\rangle \\ =\frac{-4P_{{}_{0}}^{2}\omega_{{}_{a}}^{2}\cos^{2}\beta_{0}}{H^{2}R^{2}}I_{{}_{\Pi }}^{2}\rho_{{}_{r}}^{2}\left[\nu^{2}\right.\rho_{\psi }^{2}+\omega_{{}_{0}}^{2}\rho_{{}_{\theta }}^{2}+2\nu \omega_{0}\rho_{\theta }\rho_{\psi }\sin\left.\left(\delta_{\theta }-\delta_{\psi }\right)\right];\\ \langle S\lambda \omega_{2y}\rangle ;\lambda =Q_{1}\cos\beta_{0}=\frac{4P_{0}i\omega_{a}\cos\beta_{0}I_{\Pi }}{HR}\rho_{r}\cos\left(\omega_{a}t+\left.\delta_{W}\right);\right.\\ \omega_{2y}=\nu \rho_{\phi }\cos\left(\nu t+\delta_{\phi }\right)\rho_{\theta }\sin\left(\nu t+\delta_{\theta }\right);\\ S=\frac{S^{a}}{B}=\frac{\left(2R\omega_{0}+H\cos\beta_{0}\right)}{B};\\ \langle S\lambda \omega_{2y}\rangle =\frac{4P_{0}i\omega_{a}\cos\beta_{0}I_{\Pi }\rho_{r}\left(2R\omega_{0}+H\cos\beta_{0}\right)}{HBR}\langle \nu \rho_{\theta }\rho_{\phi }\cos\left(\nu t+\delta_{\phi }\right)\sin\left(\nu t+\delta_{\theta }\right)\\ \times \cos\left(\omega_{a}t+\left.\delta_{W}\right)\right.=0;\\ \langle 2\omega_{1x}\omega_{1z}\cos2\beta_{0}\rangle =2\cos2\beta_{0}\langle \omega_{1x}\omega_{1z}\rangle =2\cos2\beta_{0}\langle \left(\dot{\theta }-\omega_{0}\psi \right)\nu \rho_{\phi }\cos\left(\nu t+\delta_{\phi }\right)\rangle \\ =2\cos2\beta_{0}\langle \left[\nu \rho_{\theta }\cos\left(\nu t+\delta_{\theta }\right)\right.-\omega_{0}\rho_{\psi }\sin\left.\left(\nu t+\delta_{\psi }\right)\right]\nu \rho_{\phi }\cos\left(\nu t+\delta_{\phi }\right)\rangle \\ =2\nu \rho_{\phi }\cos2\beta_{0}\left[\nu \rho_{\theta }\langle \cos\langle \left(\nu t+\delta_{\theta }\right)[\cos{\left(\nu t+\delta_{\phi }\right)}_{0}{}_{\psi }\sin\langle \left.\left(\nu t+\delta_{\psi }\right)\cos\left(\nu t+\delta_{\phi }\right)\right]\right.\\ =\nu \rho_{\phi }\cos2\beta_{0}\left[\nu \rho_{\theta }\cos\left(\delta_{\theta }-\delta_{\phi }\right)\right.-\omega_{0}\rho_{\psi }\sin\left.\left(\delta_{\psi }-\delta_{\phi }\right)\right];\\ \langle 2\lambda \omega_{1x}\omega_{1y}\rangle =\frac{8P_{0}i\omega_{a}\cos\beta_{0}I_{\Pi }}{HR}\rho_{r}\langle \cos\left(\omega_{a}t+\left.\delta_{W}\right)\right.\left[\nu \rho_{\theta }\cos\left(\nu t+\delta_{\theta }\right)\right.-\omega_{0}\rho_{\psi }\sin\left.\left(\nu t+\delta_{\psi }\right)\right]\\ \times \left[\nu \rho_{\psi }\cos\left(\nu t+\delta_{\psi }\right)\right.+\omega_{0}\rho_{\theta }\sin\left.\left(\nu t+\delta_{\theta }\right)\right]\rangle =0;\\ \langle -2tg2\beta_{0}\omega_{1z}\cos\beta_{0}\omega_{1y}\rangle =-2tg2\beta_{0}\cos\beta_{0}\langle \omega_{1z}\omega_{1y}\rangle \\ =-2tg2\beta_{0}\cos\beta_{0}\nu \rho_{\phi }\cos\left(\nu t+\delta_{\phi }\right)\left[\nu \rho_{\psi }\cos\left(\nu t+\delta_{\psi }\right)\right.+\omega_{0}\rho_{\theta }\sin\left.\left(\nu t+\delta_{\theta }\right)\right]\\ =-tg2\beta_{0}\cos\beta_{0}\nu \rho_{\phi }\left[\nu \right.\rho_{\psi }\cos\left(\delta_{\phi }-\delta_{\psi }\right)+\omega_{0}\rho_{\theta }\sin\left.\left(\delta_{\theta }-\delta_{\phi }\right)\right]\\ =-\nu \rho_{\phi }\cos\beta_{0}tg2\beta_{0}\left[\nu \right.\rho_{\psi }\cos\left(\delta_{\phi }-\delta_{\psi }\right)-\omega_{0}\rho_{\theta }\sin\left.\left(\delta_{\phi }-\delta_{\theta }\right)\right];\\ \langle {\dot{\omega }}_{2y}\rangle =\langle {\dot{\omega }}_{1z}\theta +\omega_{1z}\dot{\theta }\rangle =\langle \left[-\nu^{2}\right.\rho_{\phi }\cos\left(\nu t+\delta_{\phi }\right)\rho_{\theta }\sin\left(\nu t+\delta_{\theta }\right)+\nu \rho_{\phi }\cos\left(\nu t+\delta_{\phi }\right)\\ \times \nu \rho_{\theta }\cos\left.\left(\nu t+\delta_{\theta }\right)\right]\rangle =\frac{1}{2}\nu^{2}\rho_{\theta }\rho_{\phi }\left[\cos\right.\left(\delta_{\theta }-\delta_{\phi }\right)-\sin\left.\left(\delta_{\theta }-\delta_{\phi }\right)\right];\\ \langle \mu {\dot{\omega }}_{2z}\rangle =0;\\ \mu =\frac{\mu^{a}}{B}=\frac{Q\cos\beta_{0}}{B}=\frac{2P_{0}i\omega_{a}}{HBR}\left\{I_{\Pi }\right.\left[\rho_{\tau }\cos\left(\omega_{a}t+\left.\delta_{V}\right)\right.\right.\\ +\pi \rho_{r}\cos\left.\left(\omega_{a}t+\delta_{W}\right)\right]+m_{T}R_{T}L\rho_{T}\cos\left.\left(\omega_{a}t+\delta_{W_{T}}\right)\right\}. \end{array}$$

Substituting the obtained expressions in the right part of Equation (20), we find the value of the device output offset:

(35) $$\begin{array}{c} n^{2}\beta_{2}^{\left(0\right)}=-\frac{1}{2}r\nu \rho_{\phi }\rho_{\psi }\sin\left(\delta_{\psi }-\delta_{\phi }\right)-\frac{1}{4}\left[\omega_{0}\left(\rho_{\theta }^{2}+\rho_{\psi }^{2}\right)\right.-2\nu \rho_{\theta }\rho_{\psi }\sin\left.\left(\delta_{\theta }-\delta_{\psi }\right)\right]\left(q+q^{\prime }+q^{a}\right)\\ +r^{\prime }{\left[{\left(n^{2}-\nu^{2}\right)}^{2}+4{\left(h-h^{a}\right)}^{2}\nu^{2}\right]}^{-\frac{1}{2}}\left\{\nu \right.\rho_{\theta }\left[\left(r-\omega_{0}\right)\right.\nu \rho_{\theta }\cos\varepsilon -\left(r\omega_{0}-\nu^{2}\right)\rho_{\psi }\\ \times \sin\left(\delta_{\psi }-\delta_{\theta }-\varepsilon \right)-\nu q\rho_{\phi }\cos\left.\left(\delta_{\theta }-\delta_{\phi }+\varepsilon \right)\right]-\frac{1}{2}\rho_{\psi }\left[\left(r-\omega_{0}\right)\right.\nu \rho_{\theta }\sin\left(\delta_{\psi }-\delta_{\theta }+\varepsilon \right)-\left(r\omega_{0}-\nu^{2}\right)\rho_{\psi }\cos\varepsilon \\ -q\rho_{\phi }\rho_{\psi }\sin\left.\left.\left(\delta_{\psi }-\delta_{\phi }+\varepsilon \right)\right]\right\}-\frac{1}{2}\nu q^{\prime }\rho_{\phi }\left[\left(r-\omega_{0}\right)\right.\nu \rho_{\theta }\sin\left(\delta_{\phi }-\delta_{\theta }+\varepsilon \right)-\left(r\omega_{0}-\nu^{2}\right)\rho_{\psi }\\ \times \sin\left(\delta_{\psi }-\delta_{\phi }-\varepsilon \right)-q\nu \rho_{\phi }\cos\left.\varepsilon \right]{\left[{\left(n^{2}-\nu^{2}\right)}^{2}+4{\left(h-h^{a}\right)}^{2}\nu^{2}\right]}^{-\frac{1}{2}}\\ +{\left[{\left(n^{2}-\nu^{2}\right)}^{2}+4{\left(h-h^{a}\right)}^{2}\nu^{2}\right]}^{-\frac{1}{2}}\frac{R\omega_{0}\cos2\beta_{0}\cos\beta_{0}\left(2-tg2\beta_{0}tg\beta_{0}\right)-H\sin\beta_{0}}{HBR}\\ \times 2P_{0}i\omega_{a}I_{\Pi }\rho_{r}\left\{-\right.\frac{P_{0}\omega_{{}_{a}}^{2}\cos\beta_{0}}{HBR}\nu \rho_{\phi }\left[\nu \right.I_{\Pi }\rho_{\psi }\rho_{\tau }\sin\left(\delta_{W}-\delta_{V}+\varepsilon \right)\cos\left(\delta_{\phi }-\delta_{\psi }\right)\\ +\omega_{0}I_{\Pi }\rho_{\theta }\rho_{\tau }\sin\left(\delta_{W}-\delta_{V}+\varepsilon \right)\sin\left(\delta_{\theta }-\delta_{\phi }\right)+\pi \nu I_{\Pi }\rho_{r}\rho_{\psi }\sin\varepsilon \cos\left(\delta_{\phi }-\delta_{\psi }\right)\\ +\nu m_{T}R_{T}L\rho_{T}\rho_{\psi }\sin\left(\delta_{W}-\delta_{W_{T}}+\varepsilon \right)\cos\left(\delta_{\phi }-\delta_{\psi }\right)+\pi \omega_{0}I_{\Pi }\rho_{\theta }\rho_{r}\cos\varepsilon \sin\left(\delta_{\theta }-\delta_{\phi }\right)\\ +\omega_{0}m_{T}R_{T}L\rho_{\theta }\rho_{T}\cos\left(\delta_{W}-\delta_{W_{T}}+\varepsilon \right)\sin\left.\left(\delta_{\theta }-\delta_{\phi }\right)\right]\\ +\frac{2P_{0}i\omega_{a}\cos^{2}\beta_{0}I_{\Pi }\left(R\omega_{0}\sin2\beta_{0}+H\cos\beta_{0}\right)}{HBR}\nu \rho_{r}\rho_{\psi }\left[\nu \right.\rho_{\psi }\sin\varepsilon \\ +\omega_{0}\rho_{\theta }\cos\left(\delta_{\theta }-\delta_{\psi }+\varepsilon \right)+\omega_{0}\rho_{\theta }\sin\left(\frac{\pi }{4}-\delta_{\psi }+\delta_{\theta }-\varepsilon \right)\cos\left.\frac{\pi }{4}\right]\\ +\frac{P_{0}i\omega_{a}\cos\beta_{0}}{HBR}\nu^{2}\rho_{\phi }\left[\nu \right.I_{\Pi }\rho_{\tau }\rho_{\psi }\cos\left(\delta_{W}-\delta_{V}+\varepsilon \right)\cos\left(\delta_{\phi }-\delta_{\psi }\right)\\ +\nu \rho_{\psi }\rho_{T}m_{T}R_{T}L\cos\left(\delta_{W}-\delta_{W_{T}}+\varepsilon \right)\cos\left(\delta_{\phi }-\delta_{\psi }\right)+\omega_{0}I_{\Pi }\rho_{\theta }\rho_{\tau }\sin\left(\delta_{W}-\delta_{V}+\varepsilon \right)\\ \times \sin\left(\delta_{\theta }-\delta_{\phi }\right)+\omega_{0}m_{T}R_{T}L\rho_{T}\rho_{\theta }\sin\left(\delta_{W}-\delta_{W_{T}}+\varepsilon \right)\sin\left.\left.\left(\delta_{\theta }-\delta_{\phi }\right)\right]\right\}\\ +{\left[{\left(n^{2}-\nu^{2}\right)}^{2}+4{\left(h-h^{a}\right)}^{2}\nu^{2}\right]}^{-\frac{1}{2}}\left\{\frac{R\omega_{{}_{a}}^{2}\cos\beta_{0}}{HBR}\right.\nu \rho_{\phi }\left[-\right.2\omega_{a}\left(I_{{}_{\Pi }}^{2}\rho_{{}_{\tau }}^{2}\right.+\pi^{2}I_{{}_{\Pi }}^{2}\rho_{{}_{\tau }}^{2}\\ +m_{{}_{T}}^{2}R_{{}_{T}}^{2}\left.L^{2}\right)\sin\varepsilon +2\omega_{a}\pi I_{{}_{\Pi }}^{2}\rho_{r}\rho_{\tau }\sin\left(\delta_{W}-\delta_{V}-\varepsilon \right)+2\omega_{a}m_{T}R_{T}LI_{{}_{\Pi }}\rho_{T}\rho_{\tau }\sin\left(\delta_{W_{T}}-\delta_{V}-\varepsilon \right)\\ +2\omega_{a}m_{T}R_{T}L\rho_{T}\pi I_{{}_{\Pi }}\rho_{r}\sin\left(\delta_{W}-\delta_{W_{T}}-\varepsilon \right)+\left(\omega_{a}-\nu \right)I_{{}_{\Pi }}\rho_{\tau }\left(\pi I_{{}_{\Pi }}\rho_{r}\right.+m_{T}R_{T}L\left.\rho_{T}\right)\\ \times \sin\left(\delta_{V}-\varepsilon \right)+\pi I_{{}_{{}_{\Pi }}}^{2}\rho_{r}\rho_{\tau }\left(\omega_{a}+\nu \right)\sin\left(\delta_{V}-\delta_{W}-\varepsilon \right)+I_{{}_{\Pi }}\rho_{\tau }m_{T}R_{T}L\rho_{T}\left(\omega_{a}+\nu \right)\\ \times \sin\left.\left(\delta_{V}-\delta_{W_{T}}-\varepsilon \right)\right]-\frac{2P_{0}i\omega_{a}\cos^{2}\beta_{0}I_{\Pi }\left(R\omega_{0}\sin2\beta_{0}+H\cos\beta_{0}\right)}{HBR}\\ \times \left[I_{\Pi }\right.\rho_{r}\rho_{\psi }\rho_{\tau }\nu \left(\omega_{a}-\nu \right)\sin\left(\delta_{W}-\delta_{V}-\delta_{\psi }+\delta_{\phi }-\varepsilon \right)-I_{\Pi }\rho_{r}\rho_{\tau }\rho_{\theta }\omega_{0}\left(\omega_{a}-\nu \right)\\ \times \cos\left(\delta_{W}-\delta_{V}-\delta_{\theta }+\delta_{\phi }-\varepsilon \right)+I_{{}_{\Pi }}\rho_{r}\rho_{\tau }\rho_{\psi }\nu \left(\omega_{a}+\nu \right)\sin\left(\delta_{W}+\delta_{\psi }-\delta_{V}-\delta_{\phi }-\varepsilon \right)\\ -I_{{}_{\Pi }}\rho_{r}\rho_{\tau }\rho_{\psi }\omega_{0}\left(\omega_{a}+\nu \right)\cos\left(\delta_{W}-\delta_{V}+\delta_{\theta }-\delta_{\phi }-\varepsilon \right)+I_{{}_{\Pi }}\rho_{{}_{r}}^{2}\rho_{\psi }\nu \left(\omega_{a}-\nu \right)\sin\left(\delta_{\phi }-\delta_{\psi }-\varepsilon \right)\\ -\pi I_{{}_{\Pi }}\rho_{{}_{r}}^{2}\rho_{\theta }\omega_{0}\left(\omega_{a}-\nu \right)\cos\left(\delta_{\phi }-\delta_{\theta }-\varepsilon \right)+\pi I_{{}_{\Pi }}\rho_{{}_{r}}^{2}\rho_{\psi }\nu \left(\omega_{a}+\nu \right)\sin\left(\delta_{\psi }-\delta_{\phi }-\varepsilon \right)\\ -\pi I_{{}_{\Pi }}\rho_{{}_{r}}^{2}\rho_{\theta }\omega_{0}\left(\omega_{a}+\nu \right)\cos\left(\delta_{\theta }-\delta_{\phi }-\varepsilon \right)+m_{T}R_{T}L\rho_{r}\rho_{\tau }\rho_{\psi }\nu \sin\left(\omega_{a}-\nu \right)\\ \times \sin\left(\delta_{W}-\delta_{W}{}_{{}_{T}}-\delta_{\psi }-\varepsilon \right)-m_{T}R_{T}L\rho_{r}\rho_{\theta }\rho_{T}\omega_{0}\left(\omega_{a}-\nu \right)\cos\left(\delta_{W}-\delta_{W_{T}}-\delta_{\theta }-\delta_{\phi }-\varepsilon \right)\\ +m_{T}R_{T}L\rho_{r}\rho_{\psi }\rho_{T}\nu \left(\omega_{a}+\nu \right)\sin\left(\delta_{W}-\delta_{W_{T}}+\delta_{\psi }-\delta_{\phi }-\varepsilon \right)-m_{T}R_{T}L\rho_{r}\rho_{\theta }\rho_{T}\omega_{0}\left(\omega_{a}+\nu \right)\\ \times \cos\left.\left(\delta_{W}-\delta_{W_{T}}+\delta_{\theta }-\delta_{\phi }-\varepsilon \right)\right]+\frac{4P_{0}i\omega_{{}_{a}}^{2}\cos\beta_{0}}{HBR}\nu^{2}\rho_{\phi }\left[\left(I_{{}_{\Pi }}^{2}\rho_{{}_{\tau }}^{2}\right.+m_{{}_{T}}^{2}R_{{}_{T}}^{2}\left.L^{2}\rho_{T}^{2}\right)\cos\varepsilon \right.\\ +\pi I_{{}_{\Pi }}^{2}\rho_{r}\rho_{\tau }\cos\left(\delta_{V}-\delta_{W}-\varepsilon \right)\\ +m_{T}R_{T}LI_{{}_{\Pi }}\rho_{\tau }\rho_{T}\cos\left(\delta_{V}-\delta_{W}{}_{{}_{T}}-\varepsilon \right)+m_{T}R_{T}LI_{{}_{\Pi }}\rho_{\tau }\rho_{T}\cos\left(\delta_{W}{}_{{}_{T}}-\delta_{V}-\varepsilon \right)\\ +\pi m_{T}R_{T}LI_{{}_{\Pi }}\rho_{r}\rho_{T}\cos\left.\left.\left(\delta_{W}{}_{{}_{T}}-\delta_{W}-\varepsilon \right)\right]\right\}-\frac{\omega_{0}\left(-2R\omega_{0}\sin2\beta_{0}+H\cos\beta_{0}\right)}{2B}\\ \times \left[{\left(n^{2}-\nu^{2}\right)}^{2}+4{\left(h-h^{a}\right)}^{2}\nu^{2}\right]\left\{\left\{\frac{1}{2}{\left(r-\omega_{0}\right)}^{2}\nu^{2}\rho_{{}_{\theta }}^{2}-\right.\right.\frac{1}{2}q^{2}\nu^{2}\rho_{\phi }^{2}-\left(r\omega_{0}-\nu^{2}\right)\nu q\rho_{\psi }\rho_{\phi }\\ \times \sin\left(\delta_{\psi }-\delta_{\phi }\right)-\left(r-\omega_{0}\right)\left(\omega_{0}r-\nu^{2}\right)\nu \rho_{\theta }\rho_{\psi }\sin\left(\delta_{\psi }-\delta_{\theta }\right)-\left(r-\omega_{0}\right)\nu^{2}q\rho_{\theta }\rho_{\phi }\\ \times \cos\left(\delta_{\phi }-\delta_{\theta }\right)-\frac{4P_{{}_{0}}^{2}\omega_{{}_{a}}^{2}\cos^{3}\beta_{0}}{H^{2}B^{2}R^{2}}\nu \rho_{\phi }\rho_{r}i\omega_{a}I_{\Pi }\left(R\right.\omega_{0}\sin2\beta_{0}+H\cos\left.\beta_{0}\right)\\ \times \left\{I_{\Pi }\right.\rho_{\tau }\left[\nu \right.\rho_{\psi }\cos\left(\delta_{V}-\delta_{W}\right)\sin\left(\delta_{\psi }-\delta_{\phi }\right)-\omega_{0}\rho_{\theta }\cos\left(\delta_{W}-\delta_{V}-\delta_{\theta }\right)\sin\left.\delta_{\phi }\right]\\ +\pi I_{\Pi }\nu \rho_{\psi }\cos\left(\delta_{\psi }-\delta_{\phi }\right)+m_{T}R_{T}L\rho_{T}\left[\nu \right.\rho_{\psi }\cos\left(\delta_{W_{T}}-\delta_{W}\right)\cos\left(\delta_{\psi }-\delta_{\phi }\right)\\ +\omega_{0}\rho_{\theta }\sin\left(\delta_{W}-\delta_{W_{T}}\right)\cos\left.\left.\left(\delta_{\phi }-\delta_{\theta }\right)\right]\right\}-\frac{2P_{{}_{0}}^{2}i\omega_{{}_{a}}^{3}\cos^{2}\beta_{0}}{H^{2}B^{2}R^{2}}\nu^{3}\rho_{\phi }^{2}\pi I_{\Pi }\rho_{r}\left[I_{\Pi }\right.\rho_{\tau }\sin\left(\delta_{V}-\delta_{W}\right)\\ +m_{T}R_{T}L\rho_{T}\sin\left.\left(\delta_{W_{T}}-\delta_{W}\right)\right]-\frac{P_{{}_{0}}^{2}\omega_{{}_{a}}^{2}\cos^{3}\beta_{0}I_{\Pi }\left(R\omega_{0}\sin2\beta_{0}+H\cos\beta_{0}\right)}{H^{2}B^{2}R^{2}}\nu^{2}\rho_{r}\rho_{\phi }\\ \times \left[2\nu I_{\Pi }\rho_{\psi }\rho_{\tau }\sin\left(\delta_{V}-\delta_{W}\right)\cos\left(\delta_{\psi }-\delta_{\phi }\right)+2\omega_{0}I_{\Pi }\rho_{\theta }\rho_{\tau }\cos\left(\delta_{W}-\delta_{V}\right)\cos\left(\delta_{\phi }-\delta_{\theta }\right)\right.\\ +2m_{T}R_{T}L\rho_{T}\nu \rho_{\psi }\sin\left(\delta_{W_{T}}-\delta_{W}\right)\cos\left(\delta_{\psi }-\delta_{\phi }\right)+2\omega_{0}m_{T}R_{T}L\rho_{T}\rho_{\theta }\cos\left(\delta_{W}-\delta_{W_{T}}\right)\\ \times \cos\left.\left(\delta_{\phi }-\delta_{\theta }\right)\right]+\frac{P_{{}_{0}}^{2}\omega_{{}_{a}}^{4}\cos^{2}\beta_{0}}{H^{2}B^{2}R^{2}}\nu^{2}\rho_{\phi }\left[I_{{}_{\Pi }}^{2}\right.\rho_{\tau }^{2}+\pi^{2}I_{{}_{\Pi }}^{2}\rho_{r}^{2}+m_{{}_{T}}^{2}R_{{}_{T}}^{2}L^{2}\rho_{{}_{T}}^{2}\\ +\pi I_{{}_{\Pi }}^{2}\rho_{\tau }\rho_{r}\cos\left(\delta_{V}-\delta_{W}\right)+I_{\Pi }\rho_{\tau }\rho_{T}m_{T}R_{T}L\cos\left(\delta_{V}-\delta_{W_{T}}\right)+\pi I_{\Pi }\rho_{r}\rho_{T}m_{T}R_{T}L\\ \times \cos\left.\left(\delta_{W}-\delta_{W_{T}}\right)\right]-\frac{4P_{{}_{0}}^{2}\omega_{{}_{a}}^{2}\cos^{4}\beta_{0}I_{{}_{\Pi }}^{2}{\left(R\omega_{0}\sin2\beta_{0}+H\cos\beta_{0}\right)}^{2}}{H^{2}B^{2}R^{2}}\rho_{{}_{r}}^{2}\left[\nu^{2}\right.\rho_{{}_{\psi }}^{2}+\omega_{0}^{2}\rho_{\theta }^{2}\\ +2\nu \omega_{0}\rho_{\theta }\rho_{\psi }\cos\left.\left(\delta_{\theta }-\delta_{\psi }\right)\right]-\frac{P_{{}_{0}}^{2}\omega_{{}_{a}}^{2}\cos^{2}\beta_{0}}{2H^{2}B^{2}R^{2}}\nu^{4}\rho_{{}_{\phi }}^{2}\left(I_{{}_{\Pi }}^{2}\right.\rho_{\tau }^{2}+m_{{}_{T}}^{2}R_{{}_{T}}^{2}L^{2}\left.\left.\left.\rho_{{}_{T}}^{2}\right)\right\}\right\}\\ +\frac{a}{4}\left[\nu^{2}\right.\rho_{{}_{\theta }}^{2}+\omega_{0}^{2}\rho_{\psi }^{2}+2\nu \omega_{0}\rho_{\theta }\rho_{\psi }\sin\left.\left(\delta_{\theta }-\delta_{\psi }\right)+\nu^{2}\rho_{{}_{\psi }}^{2}\right]\sin2\beta_{0}-aI_{{}_{\Pi }}^{2}\rho_{r}^{2}\frac{2P_{{}_{0}}^{2}\omega_{{}_{a}}^{2}\cos^{2}\beta_{0}}{H^{2}R^{2}}\\ \times \left[\nu^{2}\right.\rho_{{}_{\psi }}^{2}+\omega_{0}^{2}\rho_{\theta }^{2}+2\nu \omega_{0}\rho_{\theta }\rho_{\psi }\sin\left.\left(\delta_{\theta }-\delta_{\psi }\right)\right]+\frac{a}{2}\nu \rho_{\phi }\cos2\beta_{0}\left[\nu \right.\rho_{\theta }\cos\left(\delta_{\theta }-\delta_{\phi }\right)\\ -\omega_{0}\rho_{\psi }\sin\left.\left(\delta_{\psi }-\delta_{\phi }\right)\right]\\ +\frac{a}{2}\nu \rho_{\phi }\cos\beta_{0}tg2\beta_{0}\left[\nu \right.\rho_{\psi }\cos\left(\delta_{\phi }-\delta_{\psi }\right)-\omega_{0}\rho_{\theta }\sin\left.\left(\delta_{\phi }-\delta_{\theta }\right)\right]-\frac{1}{2}\nu^{2}\rho_{\theta }\rho_{\phi }\\ \times \left[\cos\right.\left(\delta_{\theta }-\delta_{\varphi }\right)-\sin\left.\left(\delta_{\theta }-\delta_{\varphi }\right)\right]. \end{array}$$

### 2.3. The Second Approximation: Asynchronous Fuselage Pitch

For asynchronous oscillations, Formula (35) is significantly simplified:

$$\begin{array}{c} n^{2}\beta_{2}^{\left(0\right)}=-\frac{1}{4}\omega_{0}\left(\rho_{\theta }^{2}+\rho_{\psi }^{2}\right)\left(q+q^{\prime }+q^{a}\right)+r^{\prime }{\left[{\left(n^{2}-\nu^{2}\right)}^{2}+4{\left(h-h^{a}\right)}^{2}\nu^{2}\right]}^{-\frac{1}{2}}\left[\nu^{2}\rho_{{}_{\theta }}^{2}\left(r-\omega_{0}\right)\right.\cos\varepsilon \\ +\frac{1}{2}\rho_{{}_{\psi }}^{2}\cos\varepsilon +\frac{1}{2}\nu^{2}q^{\prime }q\rho_{{}_{\varphi }}^{2}\left.\cos\varepsilon \right]+{\left[{\left(n^{2}-\nu^{2}\right)}^{2}+4{\left(h-h^{a}\right)}^{2}\nu^{2}\right]}^{-\frac{1}{2}}\\ \times \left[\frac{2P_{0}i\omega_{a}\cos^{2}\beta_{0}I_{\Pi }\left(R\omega_{0}\sin2\beta_{0}+H\cos\beta_{0}\right)}{HBR}\nu^{2}\rho_{\psi }^{2}\rho_{r}\sin\varepsilon \right.-\frac{2P_{0}i\omega_{{}_{a}}^{3}\cos\beta_{0}}{HBR}\\ \times \left(I_{{}_{\Pi }}^{2}\rho_{{}_{\tau }}^{2}\right.+\pi^{2}I_{{}_{\Pi }}^{2}\rho_{{}_{r}}^{2}+m_{{}_{T}}^{2}R_{{}_{T}}^{2}\left.L^{2}\rho_{T}^{2}\right)\nu \rho_{\varphi }\sin\varepsilon +\frac{4P_{0}i\omega_{{}_{a}}^{2}\cos\beta_{0}}{HBR}\left(I_{{}_{\Pi }}^{2}\rho_{{}_{\tau }}^{2}\right.+m_{{}_{T}}^{2}R_{{}_{T}}^{2}\left.L^{2}\rho_{T}^{2}\right)\\ \times \nu^{2}\rho_{\varphi }\cos\left.\varepsilon \right]-\left[{\left(n^{2}-\nu^{2}\right)}^{2}+4{\left(h-h^{a}\right)}^{2}\nu^{2}\right]\frac{\left(-2R\omega_{0}\sin2\beta_{0}+H\cos\beta_{0}\right)}{2B}\\ \times \left[\frac{1}{2}{\left(r-\omega_{0}\right)}^{2}\nu^{2}\rho_{{}_{\theta }}^{2}-\frac{1}{2}q^{2}\nu^{2}\rho_{\varphi }^{2}+\frac{P_{{}_{0}}^{2}\omega_{{}_{a}}^{4}\cos^{2}\beta_{0}}{H^{2}B^{2}R^{2}}\nu^{2}\rho_{\varphi }\left(I_{{}_{\Pi }}^{2}\rho_{{}_{\tau }}^{2}\right.+\pi^{2}I_{{}_{\Pi }}^{2}\rho_{{}_{r}}^{2}+m_{{}_{T}}^{2}R_{{}_{T}}^{2}\left.L^{2}\rho_{T}^{2}\right)\right.\\ -\frac{4P_{{}_{0}}^{2}\omega_{{}_{a}}^{2}\cos^{4}\beta_{0}I_{{}_{\Pi }}^{2}{\left(R\omega_{0}\sin2\beta_{0}+H\cos\beta_{0}\right)}^{2}}{H^{2}B^{2}R^{2}}\rho_{{}_{r}}^{2}\left(\nu^{2}\right.\rho_{{}_{\psi }}^{2}+\omega_{0}^{2}\left.\rho_{\theta }^{2}\right)\\ -\frac{P_{{}_{0}}^{2}\omega_{{}_{a}}^{2}\cos^{2}\beta_{0}}{2H^{2}B^{2}R^{2}}\nu^{4}\rho_{{}_{\varphi }}^{2}\left(I_{{}_{\Pi }}^{2}\right.\rho_{\tau }^{2}+m_{{}_{T}}^{2}R_{{}_{T}}^{2}L^{2}\left.\left.\rho_{{}_{T}}^{2}\right)\right]+\frac{a}{4}\left(\nu^{2}\right.\rho_{\theta }^{2}+\omega_{0}^{2}\rho_{{}_{\psi }}^{2}+\nu^{2}\left.\rho_{{}_{\psi }}^{2}\right)\sin2\beta_{0}\\ -aI_{{}_{\Pi }}^{2}\rho_{r}^{2}\frac{2P_{{}_{0}}^{2}\omega_{{}_{a}}^{2}\cos^{2}\beta_{0}}{H^{2}R^{2}}\left(\nu^{2}\rho_{{}_{\psi }}^{2}+\omega_{0}^{2}\rho_{\theta }^{2}\right). \end{array}$$

It is of interest to determine the zero shift of the device due only to the angular oscillations of the LV body and penetrating acoustic radiation, that is, in the absence of circulation on the rocket trajectory. It is enough to put $\omega_{0}=0$ and, of course $\beta_{0}=0$, which corresponds to the absence of the input signal of the float gyroscope. Then, at synchronous pitch we have:

$$\begin{array}{c} \beta_{2}^{\left(0\right)}=\frac{H}{2c}\nu \rho_{\theta }\rho_{\psi }\sin\left(\delta_{\theta }-\delta_{\psi }\right)-\frac{1}{k^{2}}{\left[{\left(n^{2}-\nu^{2}\right)}^{2}+4h^{2}\nu^{2}\right]}^{-\frac{1}{2}}\left\{\nu^{2}\right.\rho_{\theta }\left[\nu \right.\rho_{\psi }\sin\left(\delta_{\psi }-\delta_{\theta }-\varepsilon \right)\\ -q\rho_{\varphi }\cos\left.\left(\delta_{\theta }-\delta_{\varphi }+\varepsilon \right)\right]-\frac{\rho_{\psi }^{2}}{2}\left[\nu^{2}\right.\cos\varepsilon -q\rho_{\varphi }\sin\left.\left.\left(\delta_{\psi }-\delta_{\varphi }+\varepsilon \right)\right]\right\}\\ -\frac{1}{k^{2}}{\left[{\left(n^{2}-\nu^{2}\right)}^{2}+4h^{2}\nu^{2}\right]}^{-\frac{1}{2}}\left[-\nu^{2}\rho_{\psi }\sin\left(\delta_{\psi }-\delta_{\varphi }-\varepsilon \right)-\right.\nu \rho_{\varphi }q\cos\left.\varepsilon \right]\\ +\frac{1}{k^{2}}{\left[{\left(n^{2}-\nu^{2}\right)}^{2}+4h^{2}\nu^{2}\right]}^{-\frac{1}{2}}\left\{\frac{P_{0}\omega_{{}_{a}}^{2}}{HBR}\right.\nu \rho_{\varphi }\left[-\right.2\omega_{a}\left(I_{{}_{\Pi }}^{2}\rho_{{}_{\tau }}^{2}\right.+\pi^{2}I_{{}_{\Pi }}^{2}\rho_{{}_{r}}^{2}++m_{{}_{T}}^{2}R_{{}_{T}}^{2}\left.L^{2}\right)\sin\varepsilon \\ +2\pi \omega_{a}I_{{}_{\Pi }}^{2}\rho_{r}\rho_{\tau }\sin\left(\delta_{W}-\delta_{V}-\varepsilon \right)+2\omega_{a}m_{T}R_{T}LI_{{}_{\Pi }}\rho_{T}\rho_{\tau }\sin\left(\delta_{W_{T}}-\delta_{V}-\varepsilon \right)\\ +2\pi I_{{}_{\Pi }}\rho_{r}m_{T}R_{T}L\rho_{T}\sin\left(\delta_{W}-\delta_{W_{T}}-\varepsilon \right)+\left(\omega_{a}-\nu \right)I_{{}_{\Pi }}\rho_{\tau }\left(\pi I_{{}_{\Pi }}\rho_{r}\right.+m_{T}R_{T}L\left.\rho_{T}\right)\\ \times \sin\left(\delta_{V}-\varepsilon \right)+\pi I_{{}_{{}_{\Pi }}}^{2}\rho_{r}\rho_{\tau }\left(\omega_{a}+\nu \right)\sin\left(\delta_{V}-\delta_{W}-\varepsilon \right)+\left(\omega_{a}+\nu \right)I_{{}_{\Pi }}\rho_{\tau }m_{T}R_{T}L\rho_{T}\\ \times \sin\left.\left(\delta_{V}-\delta_{W_{T}}-\varepsilon \right)\right]-\frac{2P_{0}i\omega_{a}I_{\Pi }}{BR}\left[I_{{}_{\Pi }}\right.\rho_{r}\rho_{\psi }\rho_{\tau }\left(\omega_{a}-\nu \right)\sin\left(\delta_{W}-\delta_{V}-\delta_{\psi }+\delta_{\varphi }-\varepsilon \right)\\ +\left(\omega_{a}+\nu \right)\nu I_{{}_{\Pi }}\rho_{r}\rho_{\tau }\rho_{\psi }\sin\left(\delta_{W}+\delta_{\psi }-\delta_{V}-\delta_{\varphi }-\varepsilon \right)+\nu \left(\omega_{a}-\nu \right)I_{{}_{\Pi }}\rho_{{}_{r}}^{2}\rho_{\psi }\\ \times \sin\left(\delta_{\varphi }-\delta_{\psi }-\varepsilon \right)+\pi I_{{}_{\Pi }}\rho_{{}_{r}}^{2}\rho_{\psi }\nu \left(\omega_{a}+\nu \right)\sin\left(\delta_{\psi }-\delta_{\varphi }-\varepsilon \right)+\nu \left(\omega_{a}-\nu \right)\\ \times m_{T}R_{T}L\rho_{T}\rho_{r}\rho_{\psi }\sin\left(\delta_{W}-\delta_{W_{T}}-\delta_{\psi }-\varepsilon \right)+m_{T}R_{T}L\rho_{T}\rho_{r}\rho_{\psi }\nu \left(\omega_{a}+\nu \right)\\ \times \sin\left.\left(\delta_{W}-\delta_{W_{T}}+\delta_{\psi }-\delta_{\varphi }-\varepsilon \right)\right]+\frac{4P_{0}i\omega_{{}_{a}}^{2}}{HBR}\nu^{2}\rho_{\varphi }\left[\left(I_{{}_{\Pi }}^{2}\rho_{{}_{\tau }}^{2}\right.+m_{{}_{T}}^{2}R_{{}_{T}}^{2}\left.L^{2}\rho_{T}^{2}\right)\cos\varepsilon \right.\\ +\pi I_{{}_{\Pi }}^{2}\rho_{r}\rho_{\tau }\cos\left(\delta_{V}-\delta_{W}-\varepsilon \right)+I_{{}_{\Pi }}\rho_{\tau }m_{T}R_{T}L\rho_{T}\cos\left(\delta_{V}-\delta_{W_{T}}-\varepsilon \right)\\ +I_{\Pi }\rho_{\tau }m_{T}R_{T}L\rho_{T}\cos\left(\delta_{W}{}_{T}-\delta_{V}-\varepsilon \right)+\pi I_{\Pi }\rho_{r}m_{T}R_{T}L\rho_{T}\cos\left.\left.\left(\delta_{W}{}_{T}-\delta_{W}-\varepsilon \right)\right]\right\}\\ -\frac{1}{k^{2}}aI_{{}_{{}_{\Pi }}}^{2}\rho_{r}^{2}\frac{2P_{{}_{0}}^{2}\omega_{{}_{a}}^{2}}{H^{2}R^{2}}\nu^{2}\rho_{\psi }+\frac{1}{k^{2}}\frac{a}{2}\nu^{2}\rho_{\varphi }\rho_{\theta }\cos\left(\delta_{\theta }-\delta_{\varphi }\right)-\frac{1}{2k^{2}}\nu^{2}\rho_{\theta }\rho_{\varphi }\left[\cos\right.\left(\delta_{\theta }-\delta_{\varphi }\right)\\ -\sin\left.\left(\delta_{\theta }-\delta_{\varphi }\right)\right]. \end{array}$$

In the case of asynchronous oscillations of the LV body, the zero shift of the instrument will take place only when the gyroscope is exposed to penetrating acoustic radiation. From (33), we have:

$$\begin{array}{c} \beta_{2}^{\left(0\right)}=\frac{1}{k^{2}}{\left[{\left(n^{2}-\nu^{2}\right)}^{2}+4h^{2}\nu^{2}\right]}^{-\frac{1}{2}}\left[\frac{2P_{0}i\omega_{a}I_{\Pi }}{BR}\nu^{2}\rho_{{}_{\psi }}^{2}\rho_{r}\right.\sin\varepsilon -\frac{2P_{0}\omega_{{}_{a}}^{3}}{HBR}\left(I_{{}_{\Pi }}^{2}\rho_{{}_{\tau }}^{2}\right.+\pi^{2}I_{{}_{\Pi }}^{2}\rho_{r}^{2}\\ +m_{{}_{T}}^{2}R_{{}_{T}}^{2}\left.L^{2}\rho_{T}^{2}\right)\nu \rho_{\phi }\sin\varepsilon +\frac{4P_{0}i\omega_{{}_{a}}^{2}}{HBR}\left(I_{\Pi }^{2}\rho_{\tau }^{2}+m_{T}^{2}R_{T}^{2}L^{2}\rho_{T}^{2}\right)\nu^{2}\rho_{\phi }\cos\left.\varepsilon \right]\\ -\frac{1}{k^{2}}\left[{\left(n^{2}-\nu^{2}\right)}^{2}+4h^{2}\nu^{2}\right]\frac{H}{2B}\left[-\right.\frac{1}{2}\nu^{2}q^{2}\rho_{\phi }^{2}+\frac{P_{{}_{0}}^{2}\omega_{{}_{a}}^{4}}{H^{2}B^{2}R^{2}}\nu^{2}\rho_{\phi }\left(I_{{}_{\Pi }}^{2}\rho_{{}_{\tau }}^{2}\right.+\pi^{2}I_{{}_{\Pi }}^{2}\rho_{r}^{2}\\ +m_{{}_{T}}^{2}R_{{}_{T}}^{2}\left.L^{2}\rho_{T}^{2}\right)-\frac{4P_{{}_{0}}^{2}\omega_{{}_{a}}^{2}I_{{}_{\Pi }}^{2}}{B^{2}R^{2}}\rho_{r}^{2}\left(\nu^{2}\rho_{\psi }^{2}\right)-\frac{P_{{}_{0}}^{2}\omega_{{}_{a}}^{2}}{2H^{2}B^{2}R^{2}}\nu^{4}\rho_{\phi }\left(I_{{}_{\Pi }}^{2}\rho_{{}_{\tau }}^{2}\right.+\left.m_{{}_{T}}^{2}R_{{}_{T}}^{2}\left.L^{2}\rho_{T}^{2}\right)\right]\\ -\frac{1}{k^{2}}aI_{{}_{{}_{\Pi }}}^{2}\rho_{r}^{2}\frac{2P_{{}_{0}}^{2}\omega_{{}_{a}}^{2}}{H^{2}R^{2}}\left(\nu^{2}\rho_{\psi }^{2}\right). \end{array}$$

The error in measuring the angular velocity due to the shift of zero $\beta_{2}^{\left(0\right)}$ can be determined by the formula derived from Expression (10):

(36) $$\Delta \omega \approx \frac{P_{{}_{0}}^{2}\omega_{{}_{a}}^{4}B^{2}}{H^{3}RR_{T}^{2}\beta_{2}^{\left(0\right)}}\left\{I_{\Pi }\right.\left[\rho_{\tau }\cos\right.\left(\omega_{a}t+\delta_{V}\right)+\rho_{r}\pi \cos\left.\left(\omega_{a}t+\delta_{W}\right)\right]+m_{T}R_{T}L\rho_{T}\cos\left.\left(\omega_{a}t+\delta_{W_{T}}\right)\right\}.$$

## 3. Results and Discussion

**Figure 3:**
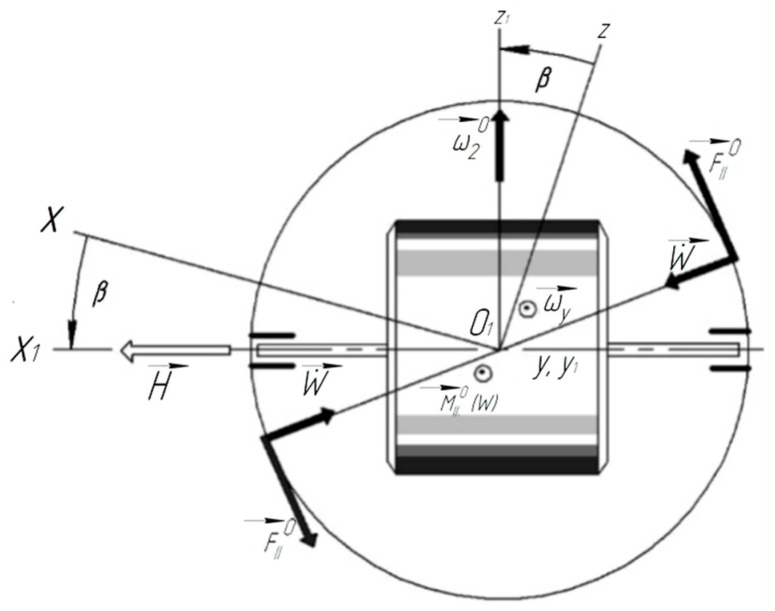
The nature of the joint action of acoustic radiation and the angular motion of the launch vehicle body on a two-degree float gyroscope.

The analysis shows that the angular velocity $\omega_{y}$ of the rocket body with acoustic vibration (sound wave) (Figure 1) of the ends of the float leads to helical motion, which in itself is a positive factor, since it reduces dry friction on the output axis. However, in combination with elastic radial displacements $\dot{W}\left(t\right)\;$ of the lateral surface of the float, the angular velocity will $\omega_{y}$ lead to the appearance of a moment of inertia forces of Coriolis and, naturally, to the appearance of an angular velocity ${\vec{\omega }}_{2}^{a}\;,\;$ a directed parallel to the input axis (sensitivity axis) of the device (Figure 3):

$$\omega_{2}^{a}=\frac{4I_{\Pi }}{HR}\left(\dot{\varphi }\sin\theta \cos\psi +\dot{\psi }\cos\theta \right)\dot{W}\left(t\right)$$

Tangential elastic displacements $V\left(t\right)$ of the lateral surface in the presence of an angular velocity $\omega_{y}$ will lead to the appearance of Coriolis inertia forces, the lines of action of which cross the center of the suspension and, thus, do not create a disturbing moment.

Let us present the scheme of sound wave impact in the form shown in Figure 1.

We take the sound pressure in the incident wave equal to

(37) $$P_{1}=P_{10}\exp i\left[\omega t-{\vec{k}}_{0}{\vec{R}}_{0}\left(z,\varphi \right)\right],$$

where $P_{10}$ is pressure in the incident wave; ${\vec{k}}_{0}=\vec{n}\frac{\omega }{c}$ is the wave vector; c is the sound speed; $\vec{n}$ is the unit vector of the wave propagation direction; and ${\vec{R}}_{0}$ is the radius vector of the float surface point.

Sound pressures can be written, taking into account the accepted designations in the diagram, also taking for simplicity the angles of incidence, reflection, and transmitted waves equal in magnitude, in the form:

(38) $$P_{1}=P_{10}\exp i\left[\omega t-k_{0}\left(R\cos\varphi \cos\varepsilon_{1}-R\sin\varphi \sin\varepsilon_{1}\cos\varepsilon_{2}-z\sin\varepsilon_{1}\sin\varepsilon_{2}\right)\right];$$

(39) $$P_{2}=P_{20}\exp i\left[\omega t-k_{0}\left(-R\cos\varphi \cos\varepsilon_{1}-R\cos\varphi \sin\varepsilon_{1}\cos\varepsilon_{2}-z\sin\varepsilon_{1}\sin\varepsilon_{2}\right)\right];$$

(40) $$P_{3}=P_{30}\exp i\left[\omega t-k_{0}\left(R\cos\varphi \cos\varepsilon_{1}-R\cos\varphi \sin\varepsilon_{1}\cos\varepsilon_{2}-z\sin\varepsilon_{1}\sin\varepsilon_{2}\right)\right]$$

The operating conditions of the aircraft adequately correspond to the diffuse structure of the sound field. In this case, the transfer of sound energy is equally probable at all angles of incidence.

The maximum values of elastic displacements of the suspension surface in the middle frame are presented in Table 1, Table 2 and Table 3.

Numerical analysis showed that the maximum elastic displacements at $P_{10}=400\;H/m^{2}$ in the direction of the length of the suspension are $0.98\times 10^{-2}\;\mu \mathrm{km}$, $5.75\times 10^{-2}\;\mu \mathrm{km}$ along the parallel and $12\times 10^{-2}\;\mu \mathrm{km}$ in the radial direction. With sound pressure values of $100\;H/m^{2}$, they are limited, respectively, $0.24\times 10^{-2}\;\mu \mathrm{km}$, $1.44\times 10^{-2}\;\mu \mathrm{km}$, and $3\times 10^{-2}\;\mu \mathrm{km}$.

If at $P_{10}=400\;H/m^{2}$ in the case of a diffuse field, the maximum values of elastic displacements along the length decreased by $0.9\times 10^{-2}\;\mu \mathrm{km}$, and in the circumferential direction by $0.4\times 10^{-2}\;\mu \mathrm{km}$, then in the transverse plane increased by $6.7\times 10^{-2}\;\mu \mathrm{km}$. In turn, at $P_{10}=100\;H/m^{2}$ they decreased by $0.23\times 10^{-2}\;\mu \mathrm{km}$ along the length and by $0.01\times 10^{-2}\;\mu \mathrm{km}$ along the parallel, but increased by $2.66\times 10^{-2}\;\mu \mathrm{km}$ in the radial direction.

**Figure 4:**
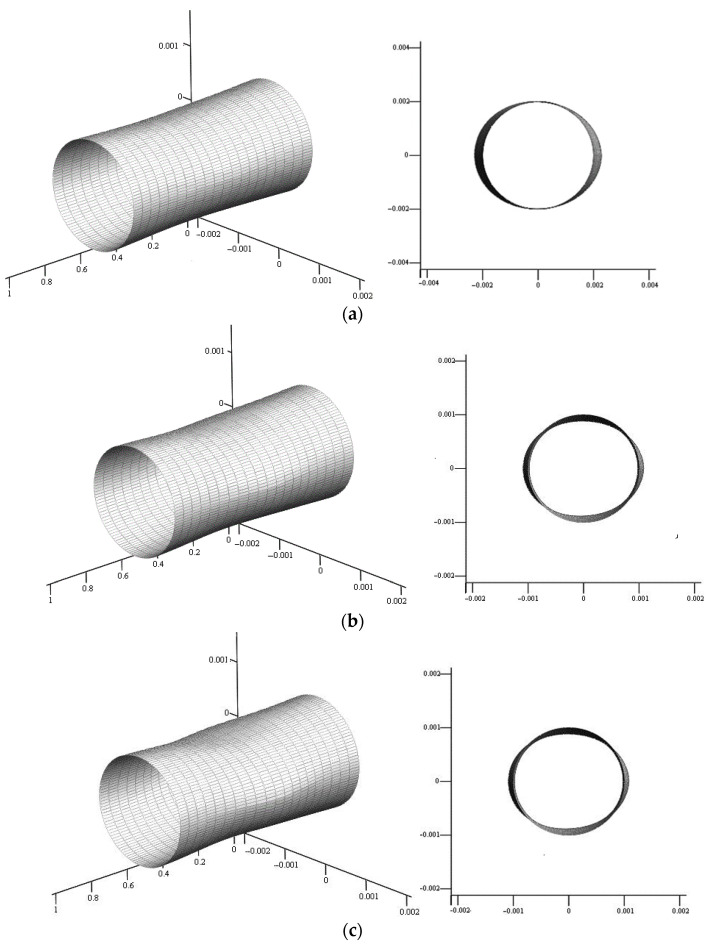

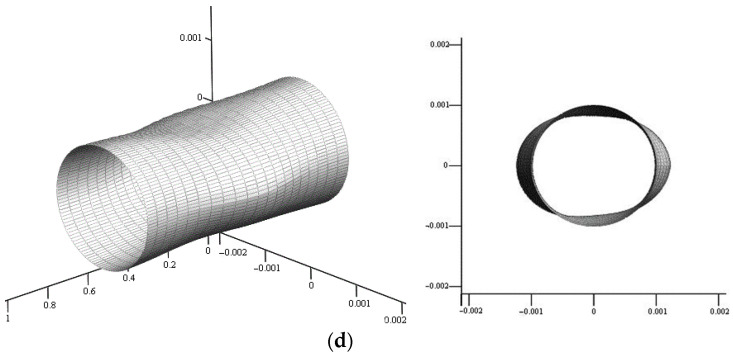
Elastically stressed state of the suspension surface in a diffuse field in axonometry and in the frontal plane: (**a**) $P_{10}=100\;H/m^{2}$**,** (**b**) $P_{10}=200\;H/m^{2}$, (**c**) $P_{10}=300\;H/m^{2}$, (**d**) $P_{10}=400\;H/m^{2}$ .

The appearance of the suspension in a diffuse field is shown in Figure 4 for four values of overpressure at radiation frequency $\omega =3000\;s^{-1}$.

In order to consider a non-stationary problem, it is necessary to introduce a factor expiωt, which will change the picture of elastic displacements, giving it a periodic character in time.

If the launch is carried out from an orbital stage (or from a mobile-based platform), it is necessary to first expand the angular velocity of its movement along the axes Oξηζ. This applies equally to the case when it becomes necessary to take into account the angular velocity of the earth’s daily rotation.

The presented results reveal the nature of the appearance of the Euler inertia forces acting on the impedance surface of the gyroscope float suspension. These forces cause additional errors in inertial sensors during flight operation.

The angular movement of the launch vehicle selects the acoustic vibration of the suspension surface, which leads to the appearance of a systematic deviation (or systematic drift) of the axis of the figure.

## 4. Conclusions

The operation of sensors that are part of navigation systems, such as gyroscopes, work in difficult conditions, which affects their accuracy. Improving the accuracy of navigation equipment will reduce fuel consumption and reduce the impact of harmful emissions on the atmosphere.

The presented results reveal the nature of the appearance of inertia forces acting on the impedance surface of the gyroscope float suspension. These forces cause additional errors in inertial sensors during flight operation.

Thus, not only synchronous but also asynchronous pitching of the fuselage can cause a systematic zero shift of the device during flight operation. Moreover, if synchronous pitching in itself generates a zero shift, then asynchronous pitching occurs only in the presence of acoustic loading of the mechanical systems of devices.

As a result, the angular motion of the launch vehicle with velocities $\omega_{x}$ and $\omega_{y}$ will contribute to the undesirable effect of acoustic vibration on the instrument readings in the form of an additional angular acceleration ${\dot{\omega }}_{1}^{a}\left(t\right)$ relative to the output axis. In turn, the angular velocity $\omega_{y}$ of the rocket body will emphasize only the radial elastic displacements of the side surface of the float $W\left(t\right)$ and thereby simulate the presence of the input value $\omega_{2}^{a}$, being in reality “false”.

When determining the error of inertial sensors of gyro-stabilized platforms during flight operation, we restrict ourselves to the consideration of synchronous and asynchronous oscillations of the fuselage, leaving aside the analysis of the effect of polyharmonic rolling. More precisely, we will carry out a numerical analysis of only the systematic component of the measurement error of a two-degree differentiating gyroscope with a liquid static suspension, as representing the greatest practical interest.

We accept the following numerical values of the quantities included in the working formula [6]: $c=0.12{\;\mathrm{Hm}}^{2}$; $B=1.01\times 10^{-4}\;{\mathrm{Hms}}^{2}$; H=0.2093 Hms; $E=7\times 10^{10}\;{\mathrm{Hm}}^{-2}$; ν=0.32; $h=1\times 10^{-4}\;m;$ L=0.06 m; $R_{0}=0.02\;m;$ $\rho =2.7\times 10^{3};$ $P_{10}=0.7\times 10^{2}\;{\mathrm{Hm}}^{2};$ $\varepsilon_{1}=\varepsilon_{2}=\frac{\pi }{6}\;\mathrm{rad};$ A=0.7; B=0.3; $I_{z}=1.5\times 10^{-4}\;{\mathrm{Hms}}^{2};$ $\nu_{1}=0.5\;s^{-1};$ $\rho_{\varphi }=\rho_{\theta }=\rho_{\psi }=\frac{\pi }{36}\;\mathrm{rad};$ n=34.5.

Thus, we first assume the presence of synchronous pitching of the fuselage, then asynchronous.

Such a simplification will introduce some error in a comparative analysis of bench tests with numerical ones, but not so much as not to ensure the confidence of the results in the realities of flight operation.

Numerical analysis shows that at an aircraft pitching frequency of 300 Hz, the systematic error of the instrument is $0.24\;{\mathrm{degs}}^{-1}$, and at a frequency of 500 Hz it is $0.35\;{\mathrm{degs}}^{-1}$. At a frequency of 600 Hz, it is $\left(-0.38\;\right)\;{\mathrm{degs}}^{-1}$; at a frequency of 800 Hz, it is $\left(-0.48\;\right)\;{\mathrm{degs}}^{-1}$; and at 960 Hz, it is $\left(-0.39\;{\mathrm{degs}}^{-1}\right)$.

The results of bench tests of industrial samples of a differentiating gyroscope showed that at a frequency of 300 Hz the systematic error is $0.24\;{\mathrm{degs}}^{-1}$, at a frequency of 500 Hz—$0.31\;{\mathrm{degs}}^{-1}$, at a frequency of 800 Hz—$\left(-0.6{\;\mathrm{degs}}^{-1}\;\right)$, and at a frequency of 960 Hz—$\left(-0.13\;{\mathrm{degs}}^{-1}\right)$.

Thus, according to the results of bench tests, the peak values of the float gyroscope error occur at acoustic radiation frequencies of 300 Hz, 500 Hz, 700 Hz, 800 Hz, and 960 Hz. Numerical analysis revealed the peak values of instrument errors, for experimental conditions, also at frequencies of 300 Hz, 500 Hz, and 800 Hz. Moreover, they practically coincided not only in size, but also in sign.

Some discrepancy occurs at frequencies of 700 Hz and 960 Hz. Bench tests at a frequency of 700 Hz reveal an error of the device in $\left(-0.27\;{\mathrm{degs}}^{-1}\right)$, and theoretically, an error in $\left(-0.35\;{\mathrm{degs}}^{-1}\right)\;$ at a frequency of 580 Hz, i.e., to the left on the frequency axis. The difference is $0.08\;{\mathrm{degs}}^{-1}$, with a sensitivity threshold of $0.09\;{\mathrm{degs}}^{-1}$, i.e., they can be neglected. Bench tests at a frequency of 960 Hz show an error of $\left(-0.14\;{\mathrm{degs}}^{-1}\right)$, and theoretical ones set its value at $\left(-0.38\;{\mathrm{degs}}^{-1}\right)$.

These inconsistencies are, first of all, quite acceptable, and secondly, they have a fairly convincing explanation. On the stand, the device is located on elastic braces, which, due to the large absorption coefficient, neutralize the influence of walls and floors on the device on the one hand, and on the other hand, the braces form a polyharmonic roll of the device, while theoretical calculations assume the presence of a deterministic synchronous roll of the fuselage, or asynchronous.

The observed effect of selectivity by the angular movement of the aircraft body of the frequencies is generated by acoustic radiation of vibrations of the surface of the float suspension, generating the elastically stressed state of the gyroscope suspension. As a consequence, the appearance of false angular velocity on the input axis and false angular acceleration on the output axis leads to a more saturated error spectrum along the frequency axis. The numerical analysis of the adopted fixation of synchronous or asynchronous hull pitching, naturally, impoverished the error spectrum. At the same time, the coincidence of the largest, peak values, errors on the stand and in the calculations was perfectly confirmed.

**Figure 5:**
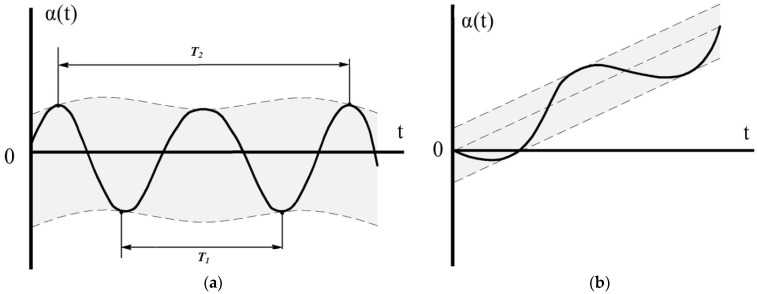
Platform drifts under the action of a shock wave: (**a**) periodic components; and (**b**) systematic and periodic components.

As a result, relative to the stabilization axes, the platform will perform not only oscillatory motion, but also have an additional systematic drift due to shock wave diffraction on the suspension of sensitive elements—two-degree gyroscopes (Figure 5).

The three-dimensional computational model did not take into account the nonlinear oscillations of the suspension ends in the acoustic field, rightly believing that the shell part is more susceptible to penetrating radiation and is the most vulnerable in terms of the tasks being solved. At the ends of the float, there are components that will significantly weaken this effect, namely, a bellows, a torque sensor, stops, etc.

Further refinement of the calculation model will make it possible to bring the theory significantly closer to the experiment, create conditions for analyzing the influence of resonance-type features in the liquid-phase part of the suspension, the nature of acoustic radiation energy dissipation in the element base, the causes of caustic zones, and the manifestation of other factors. Finally, it will allow for the choosing of technical solutions to eliminate the influence of these perturbations on the instrument error.

## Figures and Tables

**Table 1:** Maximum values of elastic displacements $U_{z\max}$ along the length of the suspension.

ω, $s^{-1}$	$U_{z}$ , m			
	$P_{10}=100,\;H/m^{2}$	$P_{10}=200,\;H/m^{2}$	$P_{10}=300,\;H/m^{2}$	$P_{10}=400,\;H/m^{2}$
600	0.2444 × 10^−8^	0.4888 × 10^−8^	0.7332 × 10^−8^	0.9776 × 10^−8^
1200	0.1665 × 10^−8^	0.333 × 10^−8^	0.4995 × 10^−8^	0.666 × 10^−8^
1800	0.09223 × 10^−8^	0.18446 × 10^−8^	0.27669 × 10^−8^	0.36892 × 10^−8^
2400	0.05072 × 10^−8^	0.10144 × 10^−8^	0.15216 × 10^−8^	0.20288 × 10^−8^
3000	0.03764 × 10^−8^	0.07528 × 10^−8^	0.11292 × 10^−8^	0.15056 × 10^−8^
3600	0.03449 × 10^−8^	0.06898 × 10^−8^	0.10347 × 10^−8^	0.13796 × 10^−8^
4200	0.03075 × 10^−8^	0.0615 × 10^−8^	0.09225 × 10^−8^	0.123 × 10^−8^
4800	0.0265 × 10^−8^	0.053 × 10^−8^	0.0795 × 10^−8^	0.106 × 10^−8^
5400	0.02361 × 10^−8^	0.04722 × 10^−8^	0.07083 × 10^−8^	0.09444 × 10^−8^
6000	0.02163 × 10^−8^	0.04326 × 10^−8^	0.06489 × 10^−8^	0.08652 × 10^−8^

**Table 2:** Maximum values of elastic displacements $U_{\varphi \max}$ along the parallel.

ω $,\;s^{-1}$	$U_{\varphi }$ , m			
	$P_{10}=100,\;H/m^{2}$	$P_{10}=200,\;H/m^{2}$	$P_{10}=300,\;H/m^{2}$	$P_{10}=400,\;H/m^{2}$
600	1.384 × 10^−8^	2.768 × 10^−8^	4.152 × 10^−8^	5.536 × 10^−8^
1200	1.405 × 10^−8^	2.81 × 10^−8^	4.215 × 10^−8^	5.62 × 10^−8^
1800	1.425 × 10^−8^	2.85 × 10^−8^	4.275 × 10^−8^	5.7 × 10^−8^
2400	1.436 × 10^−8^	2.872 × 10^−8^	4.308 × 10^−8^	5.744 × 10^−8^
3000	1.438 × 10^−8^	2.876 × 10^−8^	4.314 × 10^−8^	5.752 × 10^−8^
3600	1.437 × 10^−8^	2.874 × 10^−8^	4.311 × 10^−8^	5.748 × 10^−8^
4200	1.437 × 10^−8^	2.874 × 10^−8^	4.311 × 10^−8^	5.748 × 10^−8^
4800	1.436 × 10^−8^	2.872 × 10^−8^	4.308 × 10^−8^	5.744 × 10^−8^
5400	1.434 × 10^−8^	2.868 × 10^−8^	4.302 × 10^−8^	5.736 × 10^−8^
6000	1.432 × 10^−8^	2.864 × 10^−8^	4.296 × 10^−8^	5.728 × 10^−8^

**Table 3:** Maximum values of elastic displacements $W_{\max}$ in the plane of the middle frame.

ω $,\;s^{-1}$	W , m			
	$P_{10}=100,\;H/m^{2}$	$P_{10}=200,\;H/m^{2}$	$P_{10}=300,\;H/m^{2}$	$P_{10}=400,\;H/m^{2}$
600	3 × 10^−8^	6 × 10^−8^	9 × 10^−8^	12 × 10^−8^
1200	2.986 × 10^−8^	5.972 × 10^−8^	8.958 × 10^−8^	11.944 × 10^−8^
1800	2.971 × 10^−8^	5.942 × 10^−8^	8.913 × 10^−8^	11.844 × 10^−8^
2400	2.958 × 10^−8^	5.916 × 10^−8^	8.874 × 10^−8^	11.832 × 10^−8^
3000	2.948 × 10^−8^	5.896 × 10^−8^	8.844 × 10^−8^	11.792 × 10^−8^
3600	2.941 × 10^−8^	5.882 × 10^−8^	8.823 × 10^−8^	11.764 × 10^−8^
4200	2.933 × 10^−8^	5.866 × 10^−8^	8.799 × 10^−8^	11.732 × 10^−8^
4800	2.924 × 10^−8^	5.848 × 10^−8^	8.772 × 10^−8^	11.696 × 10^−8^
5400	2.915 × 10^−8^	5.83 × 10^−8^	8.745 × 10^−8^	11.66 × 10^−8^
6000	2.904 × 10^−8^	5.808 × 10^−8^	8.712 × 10^−8^	11.616 × 10^−8^
